# Plastome Structural Evolution and Homoplastic Inversions in Neo-Astragalus (Fabaceae)

**DOI:** 10.1093/gbe/evab215

**Published:** 2021-09-17

**Authors:** Joseph L M Charboneau, Richard C Cronn, Aaron Liston, Martin F Wojciechowski, Michael J Sanderson

**Affiliations:** 1 Department of Ecology and Evolutionary Biology, University of Arizona, Tucson, Arizona, USA; 2 Pacific Northwest Research Station, USDA Forest Service, Corvallis, Oregon, USA; 3 Department of Botany and Plant Pathology, Oregon State University, Corvallis, Oregon, USA; 4 School of Life Sciences, Arizona State University, Tempe, Arizona, USA

**Keywords:** chloroplast, inverted repeat-lacking clade, legumes, microhomology-mediated rearrangements, plastid genome

## Abstract

The plastid genomes of photosynthetic green plants have largely maintained conserved gene content and order as well as structure over hundreds of millions of years of evolution. Several plant lineages, however, have departed from this conservation and contain many plastome structural rearrangements, which have been associated with an abundance of repeated sequences both overall and near rearrangement endpoints. We sequenced the plastomes of 25 taxa of *Astragalus* L. (Fabaceae), a large genus in the inverted repeat-lacking clade of legumes, to gain a greater understanding of the connection between repeats and plastome inversions. We found plastome repeat structure has a strong phylogenetic signal among these closely related taxa mostly in the New World clade of *Astragalus* called Neo-Astragalus. Taxa without inversions also do not differ substantially in their overall repeat structure from four taxa each with one large-scale inversion. For two taxa with inversion endpoints between the same pairs of genes, differences in their exact endpoints indicate the inversions occurred independently. Our proposed mechanism for inversion formation suggests the short inverted repeats now found near the endpoints of the four inversions may be there as a result of these inversions rather than their cause. The longer inverted repeats now near endpoints may have allowed the inversions first mediated by shorter microhomologous sequences to propagate, something that should be considered in explaining how any plastome rearrangement becomes fixed regardless of the mechanism of initial formation.

**Significance**

Although the structure of most plastid (chloroplast) genomes has been remarkably conserved over evolutionary time scales, certain plant groups have had relatively frequent plastome rearrangements, including the inverted repeat-lacking clade (IRLC) of legumes. To better understand the role of repeated sequences in inversion formation, we investigated plastomes from 25 species of an IRLC genus, *Astragalus*, with and without large inversions. We found closely related species tend to resemble each other in their repeats, and plastomes with inversions do not have repeats that differ greatly overall from plastomes that do not. Specific repeated sequences are found near inversion endpoints in inverted plastomes, but we believe these repeats are there as a result of the inversions and did not cause them.

## Introduction

Nucleotide sequences, gene content, gene order, and the structure of plastid (chloroplast) genomes are highly conserved across nearly all photosynthetic green plants. Plastomes evolve at a slower rate than plant nuclear genomes (Wolfe et al. 1987; Drouin et al. 2008), and most plastomes have retained a nearly identical set of 100–120 different genes, 18 of which usually contain introns (Jansen and Ruhlman 2012). Plastome gene order has largely remained constant, as has their canonical quadripartite structure, consisting of two single-copy regions: the approximately 80-kb large single-copy region (LSC), the 20-kb small single-copy region (SSC), and the two inverted repeat (IR) regions, comprised of a single approximately 25-kb sequence present in duplicate in inverted orientation (Ruhlman and Jansen 2014). Loss of genes and major structural rearrangements are common in nonphotosynthetic plants (Wicke et al. 2013; Ruhlman and Jansen 2014), however, these are also known in several photosynthetic plant lineages. The close study of these rearrangements can shed light on the processes shaping plastome evolution, especially when examined in the plastomes of closely related taxa with and without major structural rearrangements. In this study, we examine newly sequenced plastomes in a clade within *Astragalus* L., the most species-rich genus of the inverted repeat-lacking clade (IRLC) of legumes (and of seed plants), to gain insights into a possible relationship between repeated sequences and plastome structural evolution.

Deviations from conserved sequences, gene content and order, and plastome structure have been noted in several photosynthetic plant lineages. Elevated substitution rates are known in some lineages and for certain loci (Jansen et al. 2007; Guisinger et al. 2008; Magee et al. 2010; Schwarz et al. 2017). Gene losses have also been observed, and often these have been demonstrated to result after a transfer to the nucleus (Gantt et al. 1991; Millen et al. 2001; Magee et al. 2010) or a transfer of function to nuclear-encoded genes (Ueda et al. 2008; Keller et al. 2017). Changes in gene order through large-scale inversions or expansion and contraction of the IR are noted in a number of lineages (reviewed by Downie and Palmer [1992]; Jansen and Ruhlman [2012]). For the most part inversions are thought to be rare events and have been used as phylogenetic characters (Jansen and Palmer 1987; Bruneau et al. 1990; Downie and Palmer 1992). The conservation of plastome features has been attributed to the presence of the IR (Palmer 1991), in part because high levels of large-scale inversions and other rearrangements have been noted in the few lineages in which the IR has been lost (Palmer and Thompson 1982; Palmer, Osorio, et al. 1987; Guisinger et al. 2011; Sabir et al. 2014; Sanderson et al. 2015). Nucleotide substitution rates in genes duplicated as part of the IR are also lower than in genes found in single-copy regions (Wolfe et al. 1987; Perry and Wolfe 2002; Zhu et al. 2016). Although frequent plastid genome rearrangements are known in the clades that have lost one copy of the IR (e.g., Cai et al. [2008]; Sveinsson and Cronk [2014]), many of the most highly rearranged plastomes have retained both copies such as in Campanulaceae s.l. (Haberle et al. 2008; Knox 2014), some Geraniaceae (Palmer, Nugent, et al. 1987; Chumley et al. 2006; Guisinger et al. 2011; Blazier et al. 2016), and Oleaceae (Lee et al. 2007).

The IR has been lost independently within several flowering plant families (Jansen and Ruhlman 2012) including Cactaceae (Sanderson et al. 2015), Geraniaceae (Guisinger et al. 2011; Blazier et al. 2016), Orobanchaceae (Downie and Palmer 1992; Wicke et al. 2013), and most notably Fabaceae (Lavin et al. 1990; Liston 1995), in which the loss of the IR was first observed (Kolodner and Tewari 1979) and has occurred twice independently (Lee et al. 2021). Fabaceae includes a large clade of over 4,000 species called the IRLC, defined by their absence of one copy of the IR (Wojciechowski et al. 2004) and estimated to have originated approximately 40 Ma (Lavin et al. 2005). Many plastome rearrangements have been observed in IRLC species, both ancestrally and in clades within the IRLC. Loss of genes (Gantt et al. 1991; Doyle et al. 1995; Millen et al. 2001) and introns (Jansen et al. 2008), as well as a large inversion (Doyle et al. 1996) all predate the divergence of the IRLC from its most recent common ancestor. In individual taxa or clades within the IRLC, there have been additional rearrangements observed: transfers to the nucleus (Magee et al. 2010; Sabir et al. 2014), losses of introns (Jansen et al. 2008; Sabir et al. 2014), gene duplications or partial duplications (Milligan et al. 1989; Cai et al. 2008), and many inversions (Palmer and Thompson 1982; Palmer, Osorio, et al. 1987; Milligan et al. 1989; Cai et al. 2008; Sabir et al. 2014; Choi et al. 2019). Novel IRs are even now found in the plastomes of two *Medicago* L. species after the IR was lost in an ancestor of the IRLC (Choi et al. 2019).

Repeated sequences often have been associated with rearrangements such as inversions. Specific short repeated sequences (ca. 5–30 bp) in inverted orientation have been identified at the endpoints of several plastome inversions (Hiratsuka et al. 1989; Kim et al. 2005; Chumley et al. 2006; Lee et al. 2007; Knox 2014; Schwarz et al. 2015; Wang et al. 2018) as have longer IR sequences (ca. 70–1,000 bp; Howe 1985; Lee et al. 2007; Wu et al. 2011; Guo et al. 2014). These repeats as well as sequences in tRNA genes (Hiratsuka et al. 1989; Knox et al. 1993; Hoot and Palmer 1994; Martin et al. 2014; Schwarz et al. 2015; Wang et al. 2018) have been suggested to have mediated these inversions through illegitimate recombination (Palmer, Nugent, et al. 1987; Palmer 1991). Knowledge of mechanisms of plastid DNA recombination, replication, and repair (RRR) and the genes and proteins involved has increased greatly since mechanisms of inversions were first proposed (Maréchal and Brisson 2010), and any connection sought or inferred between repeats and rearrangements must take this into account.

In plastomes with high levels of rearrangements, repeated sequences are often abundant overall and at the locations of rearrangements. This has been observed both in plastomes that have lost the IR (Milligan et al. 1989; Cai et al. 2008; Magee et al. 2010; Sabir et al. 2014; Weng et al. 2014; Choi et al. 2019) and those that have retained it (Chumley et al. 2006; Haberle et al. 2008; Guisinger et al. 2011; Knox 2014; Weng et al. 2014; Blazier et al. 2016). Multiple studies have noted highly rearranged plastomes often have elevated repeat content (Chumley et al. 2006; Cai et al. 2008; Haberle et al. 2008; Guisinger et al. 2011). Positive correlations have been found between overall repeat count and content and the number of plastome rearrangements within Geraniaceae (Weng et al. 2014), and elevated repeat counts have been noted in the vicinity of rearrangement endpoints (Sabir et al. 2014; Weng et al. 2014). Only a few studies have examined the repeated sequences present at the locations of inversion endpoints in species with and without an inversion (Kim et al. 2005; Lee et al. 2007). These were comparisons of often very distantly related species in the same family, however. Repeat content (the percentage of sites within repeats) and specific repeat sequences at inversion endpoint locations have been little explored in taxa with inversions and congeneric, closely related taxa without them.

Examining the repeat content, structure, and sequences in plastomes of closely related taxa with and without rearrangements can allow for testing specific hypotheses about the connection between repeats and rearrangements (Palmer 1991). If the presence of numerous repeats throughout the plastome makes inversions more likely through nonhomologous recombination or recombination-dependent replication, taxa with inversions might be expected to have greater overall repeat content than closely related taxa without them. Elevated repeat content at specific locations may be more important than overall repeat content in determining whether inversion take place, however, in which case taxa with inversions may have greater repeat content near inversion endpoints than taxa without them at corresponding locations. Repeated sequences may not need to be abundant for an inversion to take place if specific repeated sequences found near inversion endpoints are responsible for mediating inversions. In this case, taxa with inversions might have such sequences near or at inversion endpoints that are not present in other taxa without the inversions.

The possible association between repeats and inversions cannot be considered without examining what determines where repeated sequences are located. Repeats might have short lifespans, and the sequences themselves might form and be lost repeatedly or change position within the plastome. This has been suggested in some green algae (Pombert et al. 2006), ferns (Robison et al. 2018), and Campanulaceae s.l. (Knox 2014). If repeats are highly mutable in plastomes, then closely related taxa would not resemble each other in terms of which sequences are repeated, their locations, or their relative abundance in the plastome. Alternatively, repeat sequences and structure may not change rapidly because repeat structure is inherited over evolutionary timescales. In this case, taxa that are closely related would be expected to resemble each other (i.e., show phylogenetic signal) in repeat content and structure.

Because of the frequent rearrangements observed among the many species in the IRLC of legumes, groups within it provide an excellent opportunity to examine repeat structure and its relationship to plastome rearrangements among closely related taxa. *Astragalus* is the most species-rich genus in the IRLC (and of seed plants) with about 3,000 recognized species (Azani et al. 2019), and although almost 90 species have had plastid genomes assembled with black-box or reference-guided methods (Su et al. 2021), no previously published *Astragalus* plastome has been found to have large-scale rearrangements. However, only one species in a large clade of approximately 450–500 aneuploid *Astragalus* species endemic to North and South America called Neo-Astragalus (Wojciechowski 2005; Scherson et al. 2008) has had its plastome sequenced to date (Su et al. 2021).

During an ongoing study of the Neo-Astragalus clade, we uncovered fascinating levels of plastome structural variation that make this group an excellent study system for exploring how repeated sequences are related to the presence and placement of large-scale inversions. To this end, we sequence and assemble plastomes here from 25 *Astragalus* taxa, all but one of which is part of the Neo-Astragalus clade. We characterize and identify plastome rearrangements including changes in gene and intron content and large inversions ranging from approximately 7–40 kb in length found in four different taxa. After identifying repeats, we assess the possible phylogenetic signal in repeat-related traits, the positions of these repeats, and the repeated sequences themselves. We also seek to clarify the possible relationship between repeated sequences and inversions at multiple levels and attempt to integrate our findings from the specific rearrangements with the current knowledge of DNA replication, recombination, and repair processes in the plastome. The lability of plastome inversions and other rearrangements is also considered to address the utility of plastome inversions as phylogenetic characters.

## Results

### Plastome Assembly, Annotation, and Phylogeny

**Fig. 1.**
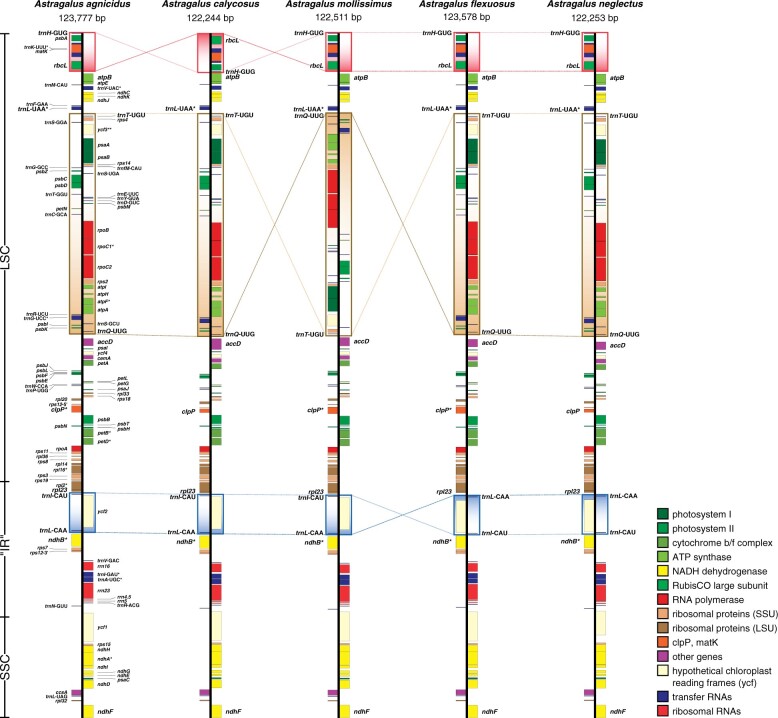
Annotated plastomes of five *Astragalus* species. Gene order in *A. agnicidus* is consistent with a plastid genome having the 50-kb inversion (Doyle et al. 1996). A 7-kb inversion is found in *A. calycosus* (*rbcL* ∼ *trnH*-GUG; red), a 40-kb inversion is found in *A. mollissimus* (*trnQ*-UUG ∼ *trnT*-UGU; tan), and 7-kb inversions are found in both *A. flexuosus and A. neglectus* (*trnL*-CAA ∼ *trnI*-CAU; blue). Inversions identified from MUMmer (Marçais et al. 2018) and progressiveMauve (Darling et al. 2010) alignments. The approximate locations of the large single-copy region (LSC), the region ancestrally duplicated as the inverted repeat but now present as a single copy only (“IR”), and the small single-copy region (SSC) are shown at the left. Plastome maps modified from the output of OGDraw (Greiner et al. 2019).

Statistics on the complete and unfragmented plastome assemblies of three *Astragalus* species sequenced at high depth and another 22 taxa sequenced at lower depth are shown in table 1. GenBank and SRA accession numbers for these annotated plastome sequences and the raw read data they were assembled from are reported in supplementary table S1, Supplementary Material online. As a member of the IRLC of legumes and thus having only one copy of the IR (fig. 1), the plastomes of *Astragalus* species are generally shorter than most, and all sequenced are between 121,590 and 124,016 bp long.

**Table 1:** Sequencing and Assembly Statistics for the Plastomes of 25 *Astragalus* Taxa

Taxon	Plastome Reads (%)	Plastome Reads (Mb)	Avg. Read Coverage	Plastome Length (bp)	GC Content (%)
*Astragalus acutirostris*	15.5	23.07	187.4×	123,082	34.14
*Astragalus agnicidus*	4.3	639.28	5,164.8×	123,777	33.97
*Astragalus americanus*	19.1	26.14	213.6×	122,398	34.29
*Astragalus ampullarioides*	17.5	29.21	237.5×	122,944	34.12
*Astragalus ampullarius*	24.0	24.31	198.3×	122,592	34.14
*Astragalus arrectus*	13.7	17.75	144.6×	122,721	34.15
*Astragalus bicristatus*	8.5	14.21	115.6×	122,963	34.11
*Astragalus bolanderi*	15.9	25.27	207.1×	122,022	34.22
*Astragalus calycosus*	17.9	32.27	264.0×	122,244	34.30
*Astragalus clevelandii*	5.8	14.52	118.3×	122,656	34.13
*Astragalus flexuosus*	5.2	732.03	5,923.6×	123,578	33.99
*Astragalus gypsodes*	34.8	35.66	291.8×	122,194	34.28
*Astragalus lentiginosus* var. *diphysus*	33.0	25.47	205.9×	123,718	34.24
*Astragalus lentiginosus* var. *mokiacensis*	17.8	25.94	209.2×	124,016	34.25
*Astragalus malacus*	32.9	43.51	353.8×	122,967	34.09
*Astragalus mollissimus*	6.1	997.82	8,144.8×	122,511	34.28
*Astragalus neglectus*	9.1	68.19	557.8×	122,253	34.14
*Astragalus nuttallianus*	41.8	45.25	368.4×	122,840	34.30
*Astragalus obscurus*	12.2	18.93	155.7×	121,590	34.21
*Astragalus pattersonii*	12.6	25.13	204.3×	122,969	34.13
*Astragalus pectinatus*	8.2	49.00	398.2×	123,069	34.07
*Astragalus serenoi*	22.6	32.64	264.5×	123,386	34.12
*Astragalus tephrodes*	14.6	22.38	182.4×	122,693	34.10
*Astragalus toanus*	6.3	9.59	78.3×	122,573	34.05
*Astragalus wootonii*	9.5	15.89	129.3×	122,915	34.11

All 25 plastomes contain the same number and complement of genes: 110 total including 76 protein-coding genes, 30 tRNAs, and four rRNAs (supplementary table S2, Supplementary Material online). Three genes (*infA*, *rpl22*, and *rps16*) and two introns (the *rps12*-3′ intron and the first *clpP* intron) found in other angiosperms are missing from all 25 plastomes. In addition, seven of 25 plastomes have lost the second *clpP* intron: those of *A. bolanderi*, *A. calycosus*, *A. malacus*, *A. neglectus*, *A. obscurus*, *A. pectinatus*, and *A. tephrodes*. There is relatively little variation in the overall percentage of sites that are protein-coding, tRNAs, and rRNAs, however one gene, *accD*, showed substantial length variation in coding sequence with several taxa having long in-frame repeats at the 5′ end of the sequence (*A. tephrodes*, *A. gypsodes*, *A. mollissimus*, *A. lentiginosus* var. *mokiacensis*, *A. lentiginosus* var. *diphysus*, and *A. wootonii*).

**Fig. 2.**
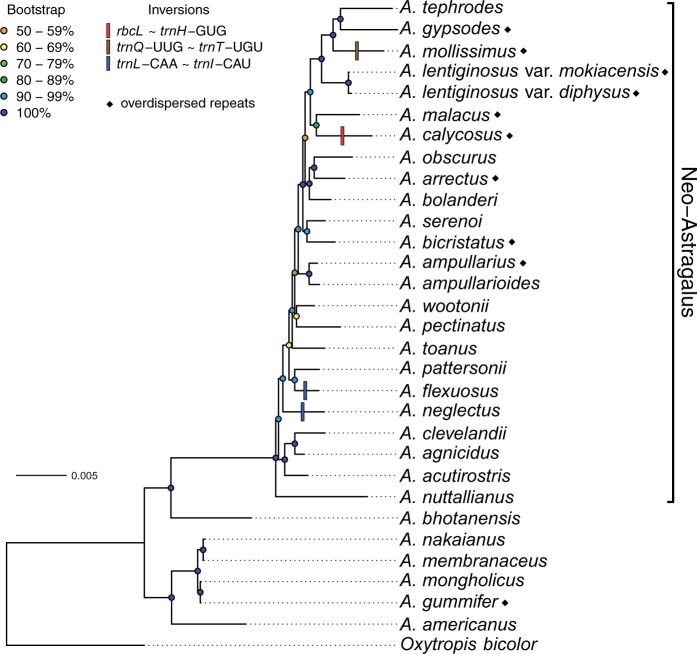
Plastome inversions and overdispersed repeats on maximum likelihood phylogram of 30 *Astragalus* taxa and *Oxytropis bicolor* from a concatenated alignment of locally colinear blocks (LCBs) identified using progressiveMauve (Darling et al. 2010). The tree is consistent with previous phylogenies of *Astragalus* at a higher level (Wojciechowski 2005; Scherson et al. 2008; Azani et al. 2019; Su et al. 2021) with Neo-Astragalus a well-supported clade nested within Old World and euploid North American taxa. Bootstrap support values from 1,000 ultrafast bootstrap replicates are shown with color-coded circles. Branch lengths are in units of substitutions per site.

The phylogeny we estimated using maximum likelihood from the newly assembled plastomes of 25 *Astragalus* taxa plus six others (see Materials and Methods) is shown in figure 2. Most clades of two to three taxa are well supported from ultrafast bootstrap replicates, though there are several relationships with relatively low bootstrap support, including one clade with only 50% bootstrap support. All newly assembled plastomes are from taxa in the Neo-Astragalus clade except euploid North American species *A. americanus*. The monophyly of Neo-Astragalus is well supported with 100% bootstrap support.

### Inversions

*Astragalus* is nested within the 50-kb inversion clade of papilionoid legumes (Doyle et al. 1996). This ancestral inversion reversed the order of genes between *matK and accD*, placing *rbcL* adjacent to *matK and rps16* adjacent to *accD*. Hereafter we refer to inversions by the two outermost loci that were moved by the inversion in the order they appear in the inverted plastome. Thus, the 50-kb inversion we would describe as *rbcL* ∼ *rps16* in the ancestor of the clade. In members of the IRLC (such as *Astragalus* spp.), which have since lost *rps16*, we refer to this inversion by the remaining outermost loci that were moved by it (*rbcL* ∼ *trnQ*-UUG). Four Neo-Astragalus plastomes contain large inversions relative to the 50-kb inversion clade gene order (fig. 1). *Astragalus calycosus* has an approximately 7-kb inversion (*rbcL* ∼ *trnH*-GUG) that placed *ndhF and rbcL* adjacent to each other at the boundary between the small and LSC regions. *Astragalus mollissimus* has an approximately 40-kb inversion (*trnQ*-UUG ∼ *trnT*-UGU) reverting much of the 50-kb inversion in the LSC, placing *trnQ*-UUG next to *trnL*-UAA and *trnT*-UGU adjacent to *accD*. Two different taxa, *A. flexuosus and A. neglectus*, have an inversion about 7 kb long (*trnL*-CAA ∼ *trnI*-CAU) that reverses the order of *trnI*-CAU, *ycf2*, and *trnL*-CAA within the former IR. The presence of the four inversions was confirmed using PCR and Sanger sequencing (see Supplementary Material online). In some taxa with inversions, weak amplification was sometimes observed from primer pairs intended to amplify when the inversion was absent in addition to strong amplification using primer pairs intended to amplify when the inversion was present (supplementary fig. S1, Supplementary Material online), indicating possible heteroplasmy for the presence of the inversions.

### Repeats

Repeat content (percentage of plastome sites within identified repeats) ranges from 2.55% in *A. bolanderi* to 3.89% in *A. tephrodes* with an average of 3.14% across all 31 taxa (table 2). The total number of nonoverlapping repeats ranges from a minimum of 41 in *A. clevelandii* to a maximum of 63 in *Oxytropis bicolor* with an average of 49.6 repeats (table 2). Repeats 30–99 bp in length (only repeats ≥30 bp were identified) comprise on an average 56.7% of the total repeat length within taxa, and repeats at least 200 bp long make up an average of just 13.2% of all sites identified as repeats within taxa (supplementary fig. S2, Supplementary Material online).

**Table 2:** Plastome Repeat Dispersion Statistics for 30 *Astragalus* Taxa and *Oxytropis bicolor*

Taxon	Repeat Content (%)	Total Repeat Count	Variance: Mean	χ^2^ Statistic	df	*P* Value	Repeat Dispersion
*Astragalus acutirostris*	2.72	42	0.90	36.10	40	0.707	Poisson
*Astragalus agnicidus*	3.29	46	1.21	48.48	40	0.336	Poisson
*Astragalus americanus*	3.23	51	0.93	36.40	39	0.822	Poisson
*Astragalus ampullarioides*	2.78	48	1.41	54.87	39	0.095	Poisson
*Astragalus ampullarius*	2.72	43	1.64	63.98	39	0.014	Overdispersed
*Astragalus arrectus*	2.65	46	1.79	69.67	39	0.004	Overdispersed
*Astragalus bicristatus*	3.17	49	1.52	59.38	39	0.039	Overdispersed
*Astragalus bolanderi*	2.55	42	1.32	51.33	39	0.179	Poisson
*Astragalus calycosus* ^a^	3.87	55	2.30	89.71	39	1.43 × 10^−5^	Overdispersed
*Astragalus clevelandii*	2.79	41	1.29	50.23	39	0.215	Poisson
*Astragalus flexuosus* ^b^	2.99	46	1.27	50.67	40	0.241	Poisson
*Astragalus gypsodes*	3.65	53	1.50	58.59	39	0.045	Overdispersed
*Astragalus lent.* var. *diphysus*	3.43	59	1.50	59.83	40	0.045	Overdispersed
*Astragalus lent.* var. *mokiacensis*	3.65	57	1.55	62.17	40	0.028	Overdispersed
*Astragalus malacus*	3.48	53	1.66	64.92	39	0.011	Overdispersed
*Astragalus mollissimus* ^c^	3.79	55	2.19	85.46	39	5.06 × 10^−5^	Overdispersed
*Astragalus neglectus* ^b^	2.77	50	1.47	57.20	39	0.060	Poisson
*Astragalus nuttallianus*	3.09	50	1.38	53.67	39	0.118	Poisson
*Astragalus obscurus*	2.67	47	1.39	54.28	39	0.106	Poisson
*Astragalus pattersonii*	3.18	49	1.33	52.00	39	0.159	Poisson
*Astragalus pectinatus*	3.32	53	1.13	45.25	40	0.524	Poisson
*Astragalus serenoi*	2.99	46	1.08	43.38	40	0.659	Poisson
*Astragalus tephrodes*	3.89	51	1.49	58.02	39	0.051	Poisson
*Astragalus toanus*	3.28	56	1.26	48.96	39	0.263	Poisson
*Astragalus wootonii*	2.74	47	1.38	53.67	39	0.118	Poisson
*Astragalus bhotanensis*	2.86	49	1.08	43.06	40	0.683	Poisson
*Astragalus gummifer*	3.19	46	1.53	61.33	40	0.033	Overdispersed
*Astragalus membranaceus*	3.10	50	1.44	57.53	40	0.072	Poisson
*Astragalus mongholicus*	2.88	45	1.46	58.55	40	0.059	Poisson
*Astragalus nakaianus*	3.10	50	1.44	57.53	40	0.072	Poisson
*Oxytropis bicolor*	3.66	63	1.27	49.38	39	0.247	Poisson

Note.—Repeat counts in nonoverlapping 3-kb windows; repeat dispersion assessed with two-tailed Pearson χ^2^ tests of deviation from the Poisson null expectation (α = 0.05).

a Inversion *rbcL* ∼ *trnH*-GUG present.

b Inversion *trnL*-CAA ∼ *trnI*-CAU present.

c Inversion *trnQ*-UUG ∼ *trnT*-UGU present.

**Fig. 3.**
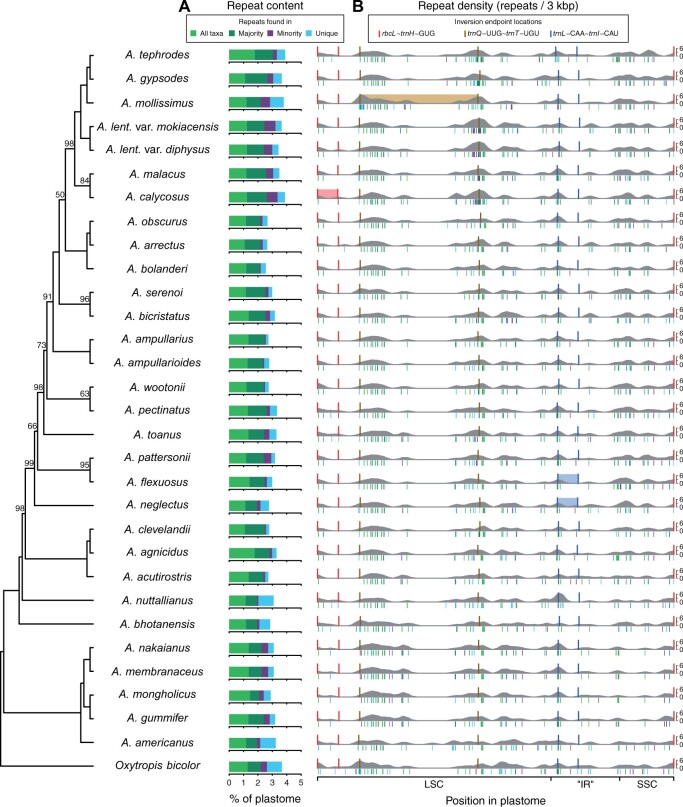
Phylogenetic context of plastome repeat content by category (*A*) and repeat density by position in plastome (*B*) for 30 *Astragalus* taxa and *Oxytropis bicolor*. Cladogram of maximum likelihood topology shown on left. Repeats are categorized by their occurrence among taxa based on Markov clustering. Repeat density in 3-kb sliding windows is averaged over 100-bp steps. The position of repeats colored by category is shown below the horizontal axis in repeat density plots. All plastomes rescaled to the same length. Inversion endpoint locations are shown in all taxa, and colored rectangles are present in taxa with inversions. All inversions were reverted before calculating repeat density.

Many of the repeats identified within single plastomes were also found in the plastomes of other taxa. Markov clustering (Van Dongen 2002) based on BLAST (Altschul et al. 1990) results placed repeats within 71 clusters. Of 1,239 total repeated sequences identified among all taxa, 927, or nearly 75% were found in at least two taxa. Ten clusters were found in all taxa, 19 clusters in the majority of taxa, and 42 clusters in a minority of taxa. The 312 repeats not placed in a cluster were unique to the taxon in which they were identified. The percentage of the sequence in each plastome identified as repeats found in all taxa, the majority of taxa, a minority of taxa, and unique to each plastome is shown in figure 3*A*. For every taxon, at least 50% of all repeat sites were part of repeats identified in all or the majority of taxa, ranging from 57.9% of repeat sites in *A. mollissimus* to 90.9% in *A. ampullarius*.

Intergenic spacers include a plurality of repeat sites in all taxa with an average of 56.9% of combined repeat length, although intergenic spacers comprise only 30.4% of plastomes on an average. Repeats in exons make up the next largest segment with an average of 37.9% of repeat length, considerably less than the average of 59.2% of all plastome sites in genes. Repeats are least often located in introns, with an average of only 5.2% across all taxa, whereas introns comprise on an average 10.4% of plastomes.

The placement of repeated sequences within plastomes appears to be conserved across all taxa in some plastomes locations and within smaller clades in other locations (fig. 3*B*). Shared regions with concentrated or sparse repeats are apparent among all plastomes. Within some clades, there also appears to be shared repeat structure. For example, repeat density (the number of repeats per 3-kb region) is especially elevated in the region near the second *trnQ*-UUG ∼ *trnT*-UGU inversion endpoint in the smallest clade that includes *A. tephrodes and A. calycosus* (fig. 3*B*, top center). Regions without repeats are also conserved. No plastome has repeats between *trnC*-GCA and *rpoC2*, a region on an average about 11.5 kb long, with one exception: *A. americanus*, which has a 33-bp repeat in this region that includes three long, closely spaced genes: *rpoB*, *rpoC1*, and *rpoC2* (central portion of the LSC in fig. 3*B*). Repeats are conspicuously sparse in much of the SSC in the smallest clade including *A. nakaianus and A. gummifer* (fig. 3*B*, lower right).

Ten *Astragalus* taxa (fig. 2) deviated from the null Poisson expectation for the dispersion of repeats across the plastome according to Pearson χ^2^ tests (table 2), that is, they had significantly greater variance in the number of repeats in 3-kb windows than expected from a Poisson distribution. The remainder of the taxa did not deviate significantly from the Poisson expectation of equal variance and mean repeat count.

Many plastome characteristics related to repeats show evidence of a strong phylogenetic signal (supplementary table S3, Supplementary Material online), including both the total length of all repeats (Pagel’s λ=0.947, *P *=* *0.044) and the percent of plastome sites that are within repeats (repeat content; λ=0.948, *P *=* *0.032). The combined length of repeats shared by all taxa does not show a phylogenetic signal (λ=0.000, *P *=* *1.000) perhaps because there is (understandably) little variation observed. We detected a strong phylogenetic signal in the length of repeats found in the majority of (but not all) taxa (λ=0.629, *P *=* *0.003), the length of repeats found in a minority of taxa (λ=0.988, *P *=* *0.036), and the length of repeats unique to a particular taxon (λ=0.973, *P *=* *0.001). The density of repeats (repeats per 3 kb) also shows strong phylogenetic signal (λ=0.996, *P *=* *0.001) as does the ratio of the variance to the mean repeat count in 3-kb windows (λ=0.999, *P *=* *0.009), the continuous trait on which the categorical repeat dispersion trait (overdispersed, etc.) is based. This can be observed in the ML phylogeny (fig. 2), in which nine of ten taxa with overdispersed repeats are found in a single clade of 14 taxa (the smallest clade that includes both *A. tephrodes and A. ampullarioides)*.

### Repeat and Inversion Locations

The four plastomes with inversions have repeats that display a greater degree of overdispersion than the repeats in plastomes without inversions (phylogenetic *t*-test, *P *=* *0.031). This appears to be driven largely by two taxa with inversions, *A. calycosus and A. mollissimus*, which both have a greater variance to mean ratio in repeat counts per 3-kb window than the plastomes of any other taxon (supplementary fig. S3, Supplementary Material online). Both *A. calycosus and A. mollissimus* are part of the previously mentioned clade containing nine of ten taxa with overdispersed repeats.

There is a significant positive correlation between overall repeat density (repeats per 3 kb) and repeat content (percentage of plastome sites in repeats; supplementary fig. S4, Supplementary Material online). Because these two metrics are correlated when taking the relatedness of taxa into account, we examined potential associations only between overall repeat content and the presence of inversions, repeat content near loci adjacent to inversion endpoints, and repeat content in the immediate vicinity of inversion endpoints. Plastomes with inversions do not have higher repeat content than plastomes without inversions when taking phylogeny into account (supplementary fig. S5, Supplementary Material online). However, of the four taxa with inversions, *A. calycosus and A. mollissimus* have greater repeat content than all but one other taxon whereas repeat content in the two taxa with the *trnL*-CAA ∼ *trnI*-CAU inversion (*A. flexuosus and A. neglectus*) is more typical of the rest of the taxa without inversions.

**Fig. 4.**
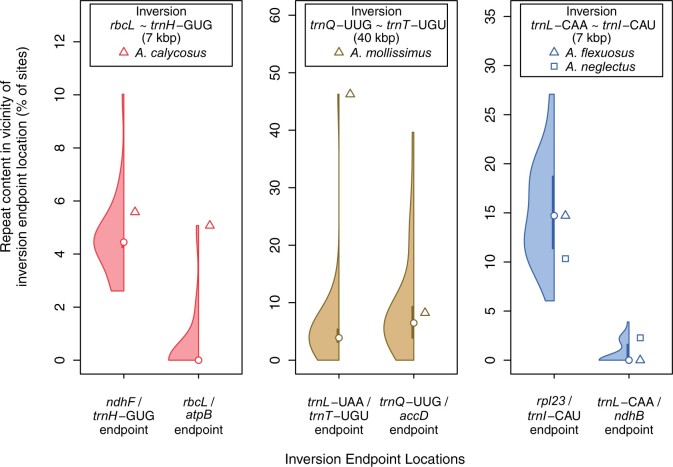
Repeat content within 1 kb of plastome inversion endpoint locations. Each endpoint location for each inversion is designated by the two loci the endpoint lies between. For each of the six inversion endpoints, on the left is a half violin plot showing the distribution of repeat content among all 31 taxa, and on the right is the repeat content at that location for the *Astragalus* taxon or taxa with the inversion. Inversions were reverted in the four taxa with them before calculating repeat content to make the two endpoints comparable with other taxa without the inversions.

Overall repeat content may have little bearing on the likelihood of an inversion occurring at specific endpoints, so we examined the repeat content in all 31 taxa within 1 kb on either side of the inversion endpoints (fig. 4). It appears that prior to the *rbcL* ∼ *trnH*-GUG inversion, repeat content was high in *A. calycosus* compared with taxa without it at the *rbcL*/*atpB* endpoint but not particularly so at the *ndhF*/*trnH*-GUG endpoint. Repeat content was especially elevated at the *trnL*-UAA/*trnT*-UGU endpoint in *A. mollissimus* before the *trnQ*-UUG ∼ *trnT*-UGU inversion, but not elevated at the *trnQ*-UUG/*accD* endpoint. For *A. flexuosus and A. neglectus* with the *trnL*-CAA ∼ *trnI*-CAU inversion, repeat content is not particularly elevated around both inversion endpoints, although repeat content at the *rpl23*/*trnI*-CAU endpoint is quite high in all taxa.

There are more repeats than expected within 1 kb of inversion endpoint locations in all plastomes regardless of whether they have an inversion or not. On an average, 5.23 repeats more repeats are found in the regions than expected from their sizes (mean 5.61 repeats). In 18 of 31 plastomes (including three of the four plastomes with inversions), there are significantly more repeats than expected from the overall frequency of repeats in the plastome according to χ^2^ goodness-of-fit tests (supplementary table S4, Supplementary Material online). Because the inversion endpoints are found in intergenic spacers, which are enriched for repeats in general, we also performed χ^2^ goodness-of-fit tests for repeat counts in the six intergenic spacers containing inversion endpoints. More repeats are found in these six spacers than expected in 29 of 31 plastomes given the frequency of repeats in intergenic spacers across whole plastomes. Eleven taxa have significantly more repeats than expected in these six spacers, including only one taxon with an inversion, *A. mollissimus* (supplementary table S5, Supplementary Material online). On an average, intergenic spacers with inversion endpoint locations contain 2.74 more repeats than expected given their length (mean 3.59 repeats).

### Specific Repeat Sequences and Inversion Endpoints

**Fig. 5.**
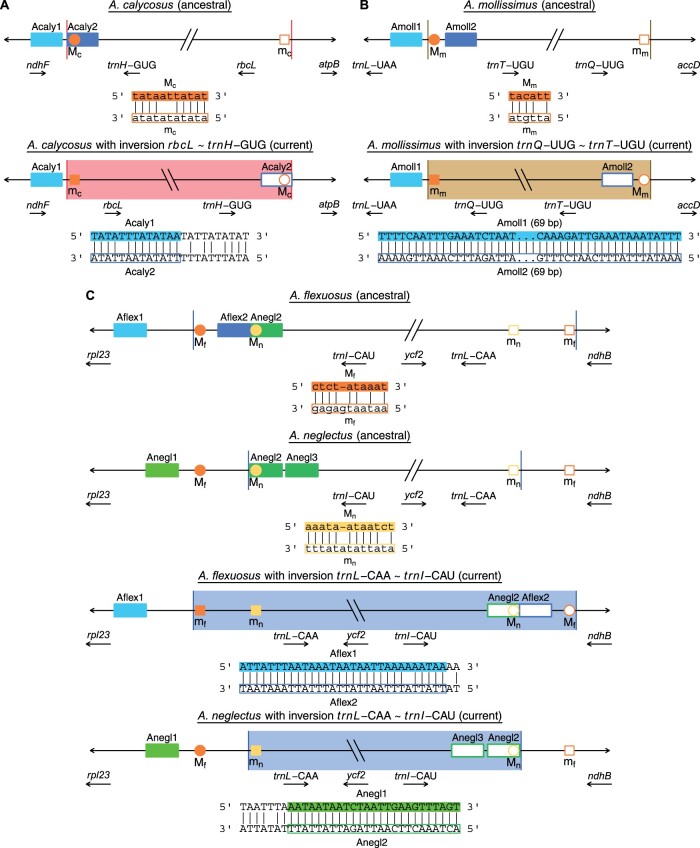
Position of repeats and microhomologous sequences ancestrally (upper) and currently (lower) after plastome inversions in *Astragalus calycosus* (*A*), *A. mollissimus* (*B*), as well as *A. flexuosus and A. neglectus* (*C*). In each case, two longer repeats (Acaly1, Acaly2, etc.) that are now in inverted orientation at opposite ends of the inversion are inferred to have ancestrally been direct repeats on the same end of the inversion. Shorter microhomologous sequences (*M*_c_, *m*_c_, etc.) are inferred to have mediated the inversion and are found at the exact inversion endpoints. Different repeats and microhomologous sequences are implicated in the inversions between the same sets of loci in *A. flexuosus and A. neglectus*. Size and position of features not to scale.

In all four plastomes with inversions, we identified short IR sequences near both endpoints of the inversions. After reverting the inversions at specific endpoint locations that minimized gaps in alignments with plastomes without the inversion (supplementary figs. S6–S8, Supplementary Material online), in all cases these two repeats appear to have been located on the same side of the inversion as direct repeats ancestrally, and plastomes without the inversions often have two such direct repeats. In the case of the *rbcL* ∼ *trnH*-GUG inversion in *A. calycosus* (fig. 5*A*), two 13-bp sequences with one mismatch are ancestrally located at the 3′ terminus of *ndhF* (Acaly1) and then just beyond (Acaly2) in the spacer between *ndhF and trnH*-GUG. At the other inversion endpoint is a sequence (*m*_c_) that complements with nine of first 11 sites of Acaly2 (*M*_c_).

For the *trnQ*-UUG ∼ *trnT*-UGU inversion found in *A. mollissimus* (fig. 5*B*), two exact 69-bp IRs (Amoll1 and Amoll2) are now found at opposite ends of the inversion but when reverted to the ancestral arrangement are direct repeats both on the *trnL*-UAA/*trnT*-UGU side of the inversion separated by about 150 bp. A 6-bp sequence located between the two ancestral direct repeats (*M*_m_) complements at five of six sites with a sequence at the other end of the inversion (*m*_m_).

The two taxa with inversion *trnL*-CAA ∼ *trnI*-CAU, *A. flexuosus and A. neglectus*, also have multiple repeats in the same direction in the reverted, ancestral alignment (fig 5*C*). However, the repeated sequences and their locations differ between the two taxa. *Astragalus flexuosus* ancestrally contained two 30-bp repeats with one mismatch (Aflex1 and Aflex2) in the same direction separated by 66 bp that contain one inversion endpoint. Three 25-bp direct repeats were ancestrally found in *A. neglectus* (Anegl1, Anegl2, Anegl3), with the inversion endpoint on that side located just before the start of Anegl2. Two different sets of sequences are found at the exact inversion endpoints in *A. flexuosus and A. neglectus*. In *A. flexuosus*, a 10-bp sequence repeated twice on the *rpl23*/*trnI*-CAU side of the reverted alignment (*M*_f_) complements the first four bases of a sequence at the other endpoint between *trnL*-CAA and *ndhB* and eight of ten sites if one base is removed from the reverted sequence on the *trnL*-CAA/*ndhB* side (*m*_f_). In *A. neglectus*, 5 bp at both ends complement each other (*M*_n_ and *m*_n_), and if one base is removed from the reverted sequence on the *trnL*-CAA/*ndhB* side (*m*_n_) then 11 of 12 bp including the ten of the first 10 sites at both endpoints would complement each other.

## Discussion

The structural variation in our newly sequenced and assembled *Astragalus* plastid genomes provides a great opportunity to compare the number and placement of repeated sequences in taxa with and without inversions. Increased plastome repeat content has been observed in species with high levels of rearrangements (Milligan et al. 1989; Chumley et al. 2006; Cai et al. 2008; Guisinger et al. 2011), and repeated sequences have been observed near the ends of inversions (Howe 1985; Hiratsuka et al. 1989; Kim et al. 2005; Chumley et al. 2006; Knox 2014; Schwarz et al. 2015; Wang et al. 2018), but repeat content overall and at inversion endpoints has not often been compared among plastomes with inversions and close relatives without them. We tested hypotheses about the relationship between repeat content and prevalence of inversions and other plastome rearrangements by examining in detail the position of repeats in closely related plastomes of 25 *Astragalus* taxa plus five previously sequenced and one outgroup.

### Changes to Plastome Gene Content, Introns, and Gene Order

All sequenced *Astragalus* plastomes share some changes relative to the ancestral gene content and order of angiosperms. They are all missing three genes lost by ancestors of the IRLC—*infA* by an ancestor of all rosids (Millen et al. 2001), *rpl22* by an ancestor of all legumes (Gantt et al. 1991), and *rps16* by an ancestor of the IRLC (as well as elsewhere within papilionoids independently; Doyle et al. 1995; Magee et al. 2010; Schwarz et al. 2015). The intron in the 3′ portion of *rps12* (the *cis*-intron) and the first *clpP* intron were also both lost somewhere near the origin of the IRLC (Jansen et al. 2008) and are missing from *Astragalus* plastomes. These two intron losses have occurred independently in legumes several times, and the *rps12*-3′ intron has been lost independently twice in Asparagales (Jansen et al. 2008). In addition, all *Astragalus* plastomes share a homologous 50-kb inversion (*rbcL* ∼ *trnQ*-UUG) that occurred in an ancestor of the large clade named for it, which includes most papilionoid legumes (Doyle et al. 1996; Cardoso et al. 2013).

Other rearrangements have occurred since the divergence of Neo-Astragalus species from a common ancestor, which is estimated to have been as little as approximately 4.4 Ma (Wojciechowski 2005; Azani et al. 2019). The second *clpP* intron was lost in seven Neo-Astragalus taxa. The loss of the second *clpP* intron is also known in *Glycyrrhiza glabra* L. (Sabir et al. 2014), *G. lepidota* Pursh, and *Tibetia liangshanensis* P.C. Li in the IRLC (Lee et al. 2021). Both *clpP* introns have also been lost in genistoid legume *Camoensia scandens* (Welw.) J.B. Gillett (Lee et al. 2021) and species of Poaceae, Onagraceae, and *Pinus* L. (Jansen et al. 2007, 2008). *Astragalus* plastomes have not experienced nearly as many gene losses as have some others in the IRLC, however. The *accD* gene has been lost in some *Trifolium* L. species (Magee et al. 2010; Sabir et al. 2014; Sveinsson and Cronk 2014) and has been lengthened with in-frame repeats (Magee et al. 2010; Gurdon and Maliga 2014) or truncated (Choi et al. 2019) in several IRLC species. Several *Astragalus* plastomes presented here have also experienced a lengthening or alternatively a truncation of the *accD* coding sequence. Novel gene losses in *Astragalus* plastomes have not been as prevalent as some other IRLC plastomes, some of which have lost *ycf4*, *psaI*, *ycf1*, or *rpl23* (Cai et al. 2008; Magee et al. 2010; Sabir et al. 2014).

We identified four species with large plastome inversions (7–40 kb): *rbcL* ∼ *trnH*-GUG in *A. calycosus*, *trnQ*-UUG ∼ *trnT*-UGU in *A. mollissimus*, *trnL*-CAA ∼ *trnI*-CAU in two taxa, *A. flexuosus and A. neglectus*. This rapid origin of plastome rearrangements has been observed among other congeneric taxa in the IRLC, which in some cases have experienced even more numerous inversions, such as in *Trifolium* (Milligan et al. 1989; Cai et al. 2008; Sveinsson and Cronk 2014), *Pisum sativum* L. (Palmer et al. 1988), *Lathyrus sativus* L. (Magee et al. 2010), *Vicia faba* L. (Palmer, Osorio, et al. 1987; Sabir et al. 2014), *Lens culinaris* Medik. (Sabir et al. 2014), and several *Medicago* species (Choi et al. 2019). Of the 12 loci adjacent to inversion endpoints found in four *Astragalus* species, all except *ndhF and trnH*-GUG have been adjacent to the endpoint of an inversion found in other genera of the IRLC at least once.

### Inversions and Repeat Content

We observed strong phylogenetic signal in many traits related to plastome repeats, and the position of repeats within the plastome is also clearly conserved. Although *Astragalus* plastome repeats show conservation among closely related species, only some have undergone large-scale inversions. These plastomes with inversions do not seem to differ appreciably from plastomes without them in overall repeat content, however. *Astragalus* plastomes with inversions also do not have significantly higher repeat content than those without them when phylogenetic relatedness is taken into account. Repeat content and the number of repeats in *Astragalus* plastomes are not nearly as high as has been reported for some other IRLC taxa, however (Cai et al. 2008; Sabir et al. 2014; Sveinsson and Cronk 2014; Choi et al. 2019). A positive correlation between repeat count or content and the number of rearrangements might only be observable when variation in both traits is greater than in *Astragalus*. *Astragalus* plastomes with inversions do seem to have repeats that are more overdispersed than repeats in plastomes without an inversion, however this pattern may be driven only by two of the four taxa with inverted plastomes. Repeat content near inversion endpoint locations is not always elevated in plastomes with inversions compared with ones without them and sometimes is actually lower in such locations when an inversion has taken place. The number of repeats near inversion endpoint locations is greater than expected in all plastomes whether or not they have an inversion, however.

Repeat content and number across the entire plastome or within a broad area around inversion endpoints seem to be less important in determining whether an inversion occurs in *Astragalus* plastomes with relatively few repeats than the presence of *specific* repeated sequences, although not necessarily in the same fashion as often thought. Previous studies have suggested inversions were sometimes mediated by short IR sequences present at either endpoint (Howe 1985; Hiratsuka et al. 1989; Chumley et al. 2006; Knox 2014; Martin et al. 2014; Schwarz et al. 2015; Wang et al. 2018). In all four *Astragalus* plastomes with inversions, we identified short inverted sequences (13–69 bp) repeated near both ends of the inversion, and in each case, the short repeat sequence was found at only one endpoint in the taxa lacking the inversions, either as a single sequence or as direct repeats. One of these IRs might have arisen stochastically through mutations or somehow been inserted in an inverted position at the opposite inversion endpoint only in the taxon with the inversion, and the presence of these short IRs could explain why only some of the taxa contain inversions. However, after examining alignments at both endpoints of the inversions, we do not believe these short IRs caused the inversions, but rather they were placed in their current positions and orientations as a *result* of the inversions.

For each *Astragalus* plastome inversion, there were short stretches of sequence adjacent to one of the two short IRs that did not appear to have been inverted because they best aligned to other taxa in their current positions in the inverted plastomes. This would imply the short IRs were not located at the exact inversion endpoints. When we adjusted the exact inversion endpoints in inverted plastomes to minimize indels in alignments at both ends of the inversions, in all cases the two short IRs appear ancestrally to have been located on the same side of the inversion (fig. 5 and supplementary figs. S6–S8, Supplementary Material online). If these inverted sequences now present in the plastomes with inversions were both adjacent to the same inversion endpoint and in the same orientation prior to the inversion, then they could not have been the sequences that mediated the inversion through nonhomologous recombination/replication mechanisms.

### Possible Inversion Mechanisms

Knowledge of the mechanisms of plastome RRR and the genes involved has developed concurrently with awareness of the physical structure of plastomes (reviewed in Maréchal and Brisson [2010]). The plastome has long been represented as a circular molecule (Kolodner and Tewari 1972), but we now know most plastome copies in actively replicating plastids are linear and often are present as head-to-tail linear concatemers or multiply branched forms (Bendich 2004; Oldenburg and Bendich 2004, 2015). Homologous recombination (HR) between linear plastome copies can occur through double-ended double-stranded break repair (DSBR) and recombination-dependent replication (RDR, also known as break-induced repair or BIR), which both require long stretches (at least 50–150 bp) of near perfect homology between two DNA strands to initiate (Maréchal and Brisson 2010). Low-fidelity mechanisms of break-induced replication or repair, however, can initiate at microhomologous sites potentially as short as 2–4 bp during microhomology-mediated break-induced replication (MMBIR; Hastings et al. [2009]) or microhomology-mediated end-joining (MMEJ; García-Medel et al. [2019]). Microhomology-mediated rearrangements have been shown to be common in *Arabidopsis thaliana* (L.) Heynh. mutants for RRR genes (Maréchal et al. 2009) and lines with expressed plastid-targeted restriction endonucleases (Sugimoto et al. 2020), but these rearrangements have also been observed at low levels in wildtype plants (Maréchal et al. 2009).

**Fig. 6.**
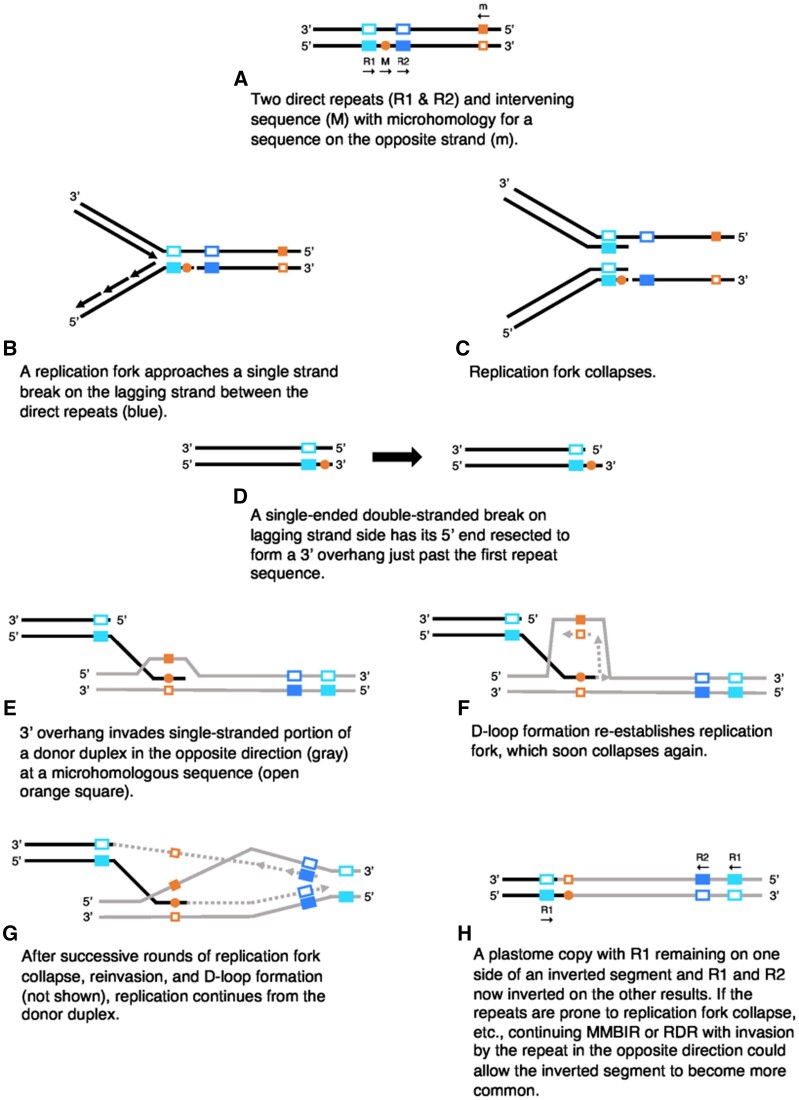
Proposed sequence of events for initiation of plastome inversions resulting in short inverted repeats at both ends from ancestrally direct repeats near one endpoint (inspired by Maréchal and Brisson [2010]). MMBIR, microhomology-mediated break-induced replication; RDR, recombination-dependent replication.

Although the short IRs at inversion endpoints in *Astragalus* plastomes appear ancestrally to have been direct repeats at one inversion endpoint only, we identified even shorter and less specific inverted sequences at the exact endpoints of each inversion that are complementary at often just the first four consecutive sites and at about 80% or more of the first 10–12 sites (fig. 5). This level of microhomology appears to be sufficient for initiating MMBIR (Hastings et al. 2009; Kwon et al. 2010; Maréchal and Brisson 2010), and we believe the inversions observed in *Astragalus* plastomes were likely initiated through MMBIR and resulted in IRs near both inversion endpoints from direct repeats that were on either side of only one of the endpoints ancestrally (fig. 6).

Our explanation for the presence of short IRs now near the endpoints of an inversion is similar to one previously proposed in grasses. Howe (1985) identified a set of 70-bp repeats near the ends of one inversion shared by many grasses that would have been inverted prior to subsequent inversions and proposed the first inversion was mediated by those IRs. Hiratsuka et al. (1989), however, believed this explanation required the pre-existence of these repeats, and proposed an alternative explanation that involved recombination between two 14-bp repeats in two tRNA genes that would explain the 70-bp repeats’ current placement.

Plastome inversions have been mediated by IRs found at both inversion endpoints ancestrally, such as the *trnS*-GCU ∼ *trnS*-GGA inversion found in multiple legumes (Martin et al. 2014; Schwarz et al. 2015; Wang et al. 2018). Often these IRs found at both endpoints prior to the inversion are longer (ca. 150–1,000 bp; Wu et al. [2011]; Guo et al. [2014]) than the IRs in *Astragalus* plastomes we have proposed were put in place by inversions (13–69 bp), and long enough to mediate HR. Having fewer repeats, especially repeats long enough for HR, might be one reason why *Astragalus* plastomes have fewer inversions than in some other IRLC genera as well as taxa in other families with highly rearranged plastomes (Haberle et al. 2008; Guisinger et al. 2011; Weng et al. 2014).

No matter the actual mechanism for producing an inversion, all mechanisms still only result in one copy of the plastome with the rearrangement, and thus for a rearrangement like an inversion to become apparently fixed at least in a single individual, the inverted copy must proliferate and the original uninverted plastome copies must be lost. If an initial inversion has an endpoint that falls between two direct repeats as we have proposed (fig. 6), this would result in short IR sequences at either end of the inversion longer than the microhomologous sequences that initiated the rearrangement. If the repeated sequence itself has properties that make replication fork collapse more likely, repeated reinitiation of replication by MMBIR at the now-inverted repeats could allow for a second reciprocal MMBIR event at the other inversion endpoint that would necessary to create an inverted plastome copy with no gene losses (Hastings et al. 2009). MMBIR is initiated with the invasion of single-stranded DNA, which is known commonly in certain contexts such as problematic DNA secondary structures (Hastings et al. 2009). Hairpin structures have also been shown to stall replication forks in bacterial, yeast, and mammalian cells (Voineagu et al. 2008).

Subsequent MMBIR or HR events mediated by now-inverted repeats could help propagate the inversion, but whether the inversion becomes fixed within a single lineage or individual (or seemingly so; there is perhaps some heteroplasmy) is dependent on genetic drift and selection. If the recombination events between short IRs—which would produce both inverted and uninverted plastome copies—cease or slow, the inverted plastome variant would have the same chance at fixation due to drift as the uninverted variant as long as they were both in equal abundance and the inversion were selectively neutral or nearly so. Demographic history (of plastids, cells, and individuals) would influence how quickly a variant is expected to become fixed through genetic drift. Small effective populations sizes or a severe bottleneck might allow a rare rearranged plastome structure to become fixed in a population over relatively short periods of time. The timing of the formation of the inversion with respect to the development of individual plants could influence the expected time to fixation for a structural variant as well. Rearranged plastomes could also become more common if they confer a fitness advantage to the plastid or the individual (e.g., by clustering functionally related genes or placing adjacent genes on the same strand), which has been proposed as a mechanism for the retention of plastome rearrangements (Cui et al. 2006). These processes that influence how abundant a plastome structural variant might become could explain why plastome inversions are found in some taxa but not in others that have the same specific repeat sequences that apparently mediated the inversion initially.

### Inversions as Phylogenetic Characters

The phylogenetic utility of inversions sometimes may be limited as we found strong evidence for independent inversions in *A. flexuosus and A. neglectus* at the same intergenic spacer regions. The slightly differing endpoints, different short inverted repeated sequences that appear to have mediated the inversions (fig. 5), and the nonsister relationship between the two species (fig. 2) suggest two independent origins of these inversions with endpoints in the same intergenic spacers. Though plastome inversions were first thought to be strong phylogenetic characters because they were not prone to homoplasy (Downie and Palmer 1992), homoplastic plastome rearrangements have now been found within at least five different angiosperm families: Ranunculaceae (Hoot and Palmer 1994), Campanulaceae (Knox 2014), Geraniaceae (Weng et al. 2014), Passifloraceae (Shrestha et al. 2019), as well as an additional examples from Fabaceae (Martin et al. 2014; Schwarz et al. 2015; Wang et al. 2018).

Although the *trnL*-CAA ∼ *trnI*-CAU inversion appears to be homoplastic for *A. flexuosus and A. neglectus*, we did find additional evidence that each of those independent inversions may be separately homologous among closer relatives of each taxon. Through PCR on additional taxa, we confirmed three other species (*A. hallii* A. Gray, *A. gracilis* Nutt., and *A. wingatanus* S. Watson) in *Astragalus* sect. *Scytocarpi* A. Gray along with *A. flexuosus* also have the same *trnL*-CAA ∼ *trnI*-CAU inversion. Another species, *A. michauxii* (Kuntze) F.J. Herm., was confirmed to have this same inversion as well. Phylogenies of Neo-Astragalus with increased taxon sampling (Charboneau JLM , Cronn RC, Liston A, Wojciechowski MF, Sanderson MJ, in preparation) indicate *A. neglectus and A. michauxii* are possible sister species and thus likely inherited this inversion from a common ancestor they did not share with sect. *Scytocarpi* species.

In addition to homoplastic inversions within families, the same intergenic spacers have been implicated in inversions even across vast evolutionary distances (Knox et al. 1993; Downie and Palmer 1994; Hoot and Palmer 1994), and the same genes and introns have also been lost independently multiple times during seed plant evolution (Jansen et al. 2007; Jansen and Ruhlman 2012). Why have these rearrangements occurred independently at common locations? If specific repeated sequences are related to the formation or retention of an inversion, then the inheritance of these sequences and their locations in the plastome could explain the common locations of rearrangements. The phylogenetic signal in repeat structure in *Astragalus* plastomes may date from a much older common ancestor shared with a larger clade of plants. Similar repeat sequences at shared locations in the plastomes of *Medicago*, *Lotus* L., *Glycine* L., and *Arabidopsis* Heynh. (Saski et al. 2005) would seem to support this idea, although the extent to which repeat structure might be conserved among even more distantly related taxa awaits future study.

Selective pressures on the plastome to maintain photosynthetic efficiency also seem to be relatively constant across autotrophic plants. Inversions and other rearrangements might be found simply where they are tolerated, and these locations may be the same across distantly related plants. Shared operons that are rarely if ever broken up by inversions (Jansen and Ruhlman 2012), and elevated substitution rates for some loci near common rearrangement locations (Magee et al. 2010; Schwarz et al. 2017) may be indicative of this. The accumulation of repeats around common locations for inversion endpoints, as we have seen in several *Astragalus* plastomes, could be symptomatic of these same selection pressures. Lengthy repeats or low-complexity sequences in general might only be allowed to accumulate or persist in plastome regions that would not be highly deleterious to break up via an inversion mediated by such repeats.

### Future Work

As more plastomes are sequenced and we gain greater insight into connections between repeated sequences and rearrangements, we must acknowledge that findings are potentially influenced by how reliably repeats and rearrangements are detected from short-read sequencing and assembly. Repeated sequences and rearrangements pose challenges to plastome assembly, and the choice of reference in reference-guided assembly or de novo assembly using sets of reads that map to references may bias against the detection of novel rearrangements. Plastome rearrangements are also not necessarily fixed within single taxa (Gurdon and Maliga 2014; Choi et al. 2020) and may not be fixed within single individuals sampled (Guo et al. 2014). Other forms of heteroplasmy, though not considered in this study, have been observed in an *Astragalus* plastome (Lei et al. 2016) as well. Long-read sequencing has begun to reveal many rearrangements are not fixed within a single individual (Ruhlman et al. 2017), and our PCR results indicate that both orientations of plastome inversions may also exist in individuals with unambiguously inverted assemblies. This is consistent with the view we have presented here, however, that a single event producing one inverted plastome copy does not alone determine whether the inversion becomes fixed in an individual, population, or taxon. Future work with long-read sequencing and sampling of multiple individuals per taxon will shed further light on the level of plastome structural variation within multiple levels of organization.

## Materials and Methods

### Sampling, DNA Extraction, and Sequencing

Plant material for DNA extraction and sequencing was collected in the field or from herbarium specimens. Collection and voucher information for the samples sequenced along with taxonomic authorities are included in supplementary table S6, Supplementary Material online. We extracted total DNA from young leaf tissue dried in silica gel or sampled from herbarium sheets using a CTAB and chloroform protocol with an RNAse A (Thermo Scientific, Waltham, MA) digestion.

Three samples (*Astragalus agnicidus*, *A. flexuosus*, and *A. mollissimus*) had whole genomic libraries prepared with Illumina TruSeq DNA library preparation kits (San Diego, CA) at the facilities in which they were sequenced. Each sample was sequenced on a single lane on the Illumina HiSeq System (San Diego, CA) with 2 × 100 bp paired-end reads. *Astragalus agnicidus* was sequenced on a HiSeq 2000 instrument at the Oregon State University Center for Genome Research and Biocomputing (CGRB), and *A. flexuosus and A. mollissimus* were sequenced on a HiSeq 2500 instrument at the Arizona State University Genomics Facility.

For all other samples, we prepared whole genomic libraries using the NEBNext Ultra II DNA Library Prep Kit for Illumina with Sample Purification Beads (New England Biolabs, Ipswich, MA) using half the volumes provided in the protocol and the option for no size selection with purified whole genomic DNA fragmented with a Bioruptor Pico sonicator (Diagenode, Denville, NJ) at OSU CGRB. Adaptor-ligated fragments were amplified with NEBNext Dual Index Primers (New England Biolabs, Ipswich, MA) with either six or eight PCR cycles. Details of library preparation and pooling are included in the Supplementary Material online. Libraries from the 25 samples reported on here were sequenced with another 71 libraries on a single midoutput lane of Illumina NextSeq 500 (San Diego, CA) with 2 × 75 bp paired-end reads at the ASU Genomics Facility.

### Sequence Data Preprocessing and Plastome Assembly

Adaptor and quality filtering of Illumina HiSeq reads from the three initial samples was performed using Trimmomatic v. 0.33 (Bolger et al. 2014) before de novo assembly with Ray v. 2.3.1 (Boisvert et al. 2010) on subsets of reads. Single contigs containing the complete plastid genome from one assembly were selected for each sample (see Supplementary Material online for details).

For the libraries sequenced with NextSeq, we processed the reads with a custom workflow using tools from the BBMap suite v. 38.12 (Bushnell B, https://sourceforge.net/projects/bbmap, last accessed July 13, 2018). Contamination- and quality-filtered reads that mapped to our three HiSeq-sequenced reference plastomes plus three NCBI RefSeq plastomes were used to assemble a preliminary plastome for each sample using the tadpole assembler of BBMap. Reads were mapped to the tadpole assembly for each sample to get a final pool of plastome reads (see Supplementary Material online for more details). Expected coverage of the plastome reads was normalized to an approximate total read depth of 100× when possible before de novo plastome assembly using SPAdes v. 3.13.0 (Bankevich et al. 2012). Otherwise all plastome reads were used in assembly. Only samples for which the entire plastome was assembled into a single contig or scaffold were used in this study.

We calculated average assembly read coverage by mapping all processed reads to the final assemblies using BBMap (Bushnell B, https://sourceforge.net/projects/bbmap, last accessed July 13, 2018). We also calculated the average read coverage in 3,000-bp windows with a step size of 100 bp using BEDtools v. 2.29.2 (Quinlan and Hall 2010) and plotted this using R v. 3.6.3 (R Core Team 2020) to examine read coverage across the length of each assembly to ensure no novel large IR had formed as it has in two *Medicago* species (Choi et al. 2019).

### Plastome Annotation

We first developed curated annotations of the three reference plastomes assembled from HiSeq (*A. agnicidus*, *A. flexuosus*, and *A. mollissimus*). These curated annotations were based on three different annotations, one using the GeSeq v. 1.8.2 webservice (https://chlorobox.mpimp-golm.mpg.de/geseq.html, last accessed July 5, 2020; Tillich et al. [2017]), and the second and third using PGA (Qu et al. 2019) with two different annotated reference plastomes, *Amborella trichopoda* Baill. (NC_005086) and *Cicer arietinum* L. (NC_011163), separately. Annotations from all three methods were checked against the annotated RefSeq plastomes of six legumes and four nonlegumes (see Supplementary Material online) and adjusted manually in Geneious v. 9.1.8 (Kearse et al. 2012) for start and stop codon positions, exon boundaries, and tRNA boundaries. We also removed annotations of non-ORF fragments of genes lost by an ancestor of all *Astragalus* species (*infA*, *rpl22*, *rps16*).

The remaining plastomes were annotated using the first three curated plastome annotations as references with PGA (Qu et al. 2019). Once again, features were examined and adjusted by hand using Geneious (Kearse et al. 2012). Gene and intron losses were noted from the output log of PGA. Several taxa had truncated *accD* annotations on the 5′ end, which we then expanded after finding an open reading frame was maintained upstream of the originally annotated start codon.

### Plastome Phylogeny Estimation

In addition to the 25 *Astragalus* plastomes sequenced and assembled here, we added six additional taxa to our phylogenetic analyses from plastome sequences available on NCBI RefSeq or GenBank: *A. bhotanensis* Baker (NC_047381), *A. gummifer* Labill. (NC_047251), *A. membranaceus* Fisch. ex Bunge (KX255662), *A. mongholicus* Bunge (NC_029828), *A. nakaianus* Y.N. Lee (NC_028171), and *Oxytropis bicolor* Bunge (NC_047482). We identified locally colinear blocks (LCBs) in each plastome using command-line progressiveMauve (Darling et al. 2010) with default settings, and extracted aligned sequences for each of the seven LCBs with sequences from all taxa. The full alignments of each LCB were concatenated to form a character matrix with 143,137 sites, 3,956 of which were parsimony-informative and 7,489 autapomorphic.

We used IQ-TREE v. 2.0.3 (Minh et al. 2020) to estimate a phylogeny using maximum likelihood from a concatenated alignment of all Mauve LCBs. See Supplementary Material online for details of model selection and estimation of bootstrap support.

### Identification and Confirmation of Inversions

Plastome inversions were identified using MUMmer v. 4.0.0 (Marçais et al. 2018) by aligning each plastome against our *A. agnicidus* reference plastome as well as with progressiveMauve (Darling et al. 2010) as described above. We also identified the locations of all inversion endpoints observed in every plastome (even when the inversion was absent) by aligning each to the plastomes identified with inversions using MUMmer. The presence of inversions identified from assemblies was confirmed using PCR (see Supplementary Material online).

### Repeat Identification and Distribution

We identified repeated sequences at least 30 bp long in plastomes with the same strategy as Choi et al. (2019) from the results of BLAST v. 2.9.0+ (Altschul et al. 1990) and Tandem Repeats Finder (TRF; Benson 1999). Details of repeat identification are can be found in the Supplementary Material online. Overlapping BLAST- and TRF-identified repeats were merged using BEDtools (Quinlan and Hall 2010), and then grouped using a custom Perl script to include all overlapping and associated (through dispersed, direct, and IRs) repeats in single groups.

To assess shared repeats among plastomes, we BLASTed one sequence from each repeat group from all taxa to each other and identified clusters of repeats using MCL v. 14.137 (Van Dongen 2002). See Supplementary Material online for details. Repeat clusters were divided into three categories by their taxon occupancy: repeat clusters found in all taxa, a majority of taxa (but not all), and a minority of taxa. A fourth category of repeats included those unique to each plastome (repeats not placed into a cluster with repeats from any other taxon). Repeats were also classified into length categories using R (R Core Team 2020), and repeat content within different parts of the plastome (protein-coding, intergenic spacers, etc.) was determined using BEDtools (Quinlan and Hall 2010).

To identify plastomes with potentially overdispersed (closer together than expected) or underdispersed (more evenly spaced than expected) repeats, we counted the number of repeats within nonoverlapping 3-kb windows using BEDtools (Quinlan and Hall 2010). To determine whether the distribution of repeats at this scale differed significantly from the Poisson expectation of variance being equal to the mean we used Pearson χ^2^ goodness-of-fit tests (two-tailed test, α = 0.05) with the sum of squared deviations of the counts in each window from the mean count (Payne et al. 2018).

We also assessed the number of repeats and repeat content in the vicinity of each inversion endpoint feature (identified with MUMmer). To maintain accurate statistics for loci at the ends of the linear representation of the plastome, we padded gene, inversion endpoint, and repeat features by appending features from the first 3 kb to the end, and prepending features from the final 3 kb to the beginning. To assess the density of repeat features across the length of plastomes in a comparable way, we first reverted repeat features within inversions when they were present using the identified inversion endpoints locations. We then tabulated repeat count and content within 1 kb of inversion endpoints using BEDtools (Quinlan and Hall 2010). We also counted repeats in sliding 3-kb windows with a step size of 100 bp from the padded, reverted repeat features as we did for the nonoverlapping windows. The density of repeat counts per 3 kb was calculated by averaging the counts in each of the windows overlapping in each 100-bp segment. The padded features added to account for the continuous nature of plastome copies (often represented as circular) were removed prior to plotting the repeat density over the rescaled length of each plastome using R (R Core Team 2020). We used χ^2^ goodness-of-fit tests to assess whether the number of repeats within 1 kb of inversion endpoint locations in each plastome was significantly greater than expected given the distributions of repeats plastome-wide. We also conducted χ^2^ tests on each plastome to determine if the six intergenic spacers containing an inversion in at least one taxon had significantly more repeats for their length than expected given the distribution of repeats across all intergenic spacers.

### Comparative Method Tests of Repeat Distribution and Inversion Status

We estimated phylogenetic signal in a number of plastome characters using Pagel’s (1999) λ with R (R Core Team 2020) (see Supplementary Material online for details). Traits examined were plastome length, total repeat length, the length of repeats from the four repeat categories (all, majority, minority, unique), total repeat content (percentage of plastome sites in repeats), repeat density (repeats per 3 kb), and the variance to mean ratio of repeat counts in 3-kb windows. We also completed a phylogenetic *t*-test to determine whether plastomes with an inversion have repeats that are more overdispersed than repeats in plastomes without an inversion based on the same variance to mean ratio of repeat counts in 3-kb windows. After using phylogenetic least squares regression (PGLS) to determine if there was a correlation between repeat content and the repeat density using R (R Core Team 2020), we also performed another phylogenetic *t*-test to assess whether plastomes with inversions have greater repeat content than plastomes without them. See Supplementary Material online for details of PGLS and phylogenetic *t*-tests. All trait values were log-transformed prior to analysis.

### Specific Repeat Sequences at Inversion Endpoint Locations

We examined the sequences around the inversion endpoint locations identified by MUMmer (Marçais et al. 2018) to pinpoint the exact location of the inversion endpoints in plastomes with inversions. In investigating this, we reverted the inversions at specific endpoint locations, extracted sequences from within 250 bp on either side of the MUMmer inversion endpoint locations from selected uninverted plastomes and the reverted plastomes and then aligned them with Geneious (Kearse et al. 2012). The exact locations of inversion endpoints were identified to minimize the number of gaps or poorly aligned regions in the alignments with uninverted plastomes for both endpoints. Repeats identified as described above were examined in the alignment regions and repeats shorter than 30 bp were also identified using the Find Repeats function of Geneious with a minimum length of 10 bp and up to 10% mismatch rate.

## Supplementary Material

evab215_Supplementary_Data
