# Supplementary material for: Plastome Structural Evolution and Homoplastic Inversions in Neo-Astragalus (Fabaceae)
Source: Genome Biol Evol. 2021 Sep 17;13(10):evab215. doi: 10.1093/gbe/evab215 (PMC8486006; doi:10.1093/gbe/evab215)
Supplement: evab215_Supplementary_Data [file evab215_supplementary_data.zip › Charboneau_et_al_GBE_supplement.pdf]

Supplementary Results

The presence of the plastome inversions found in four taxa (*rbcL* ~ *trnH*-GUG in *Astragalus calycosus*, *trnQ*-UUG ~ *trnT*-UGU in *A. mollissimus*, and *trnL*-CAA ~ *trnI*-CAU in *A. flexuosus* and *A. neglectus*) was confirmed using PCR and Sanger sequencing (see Supplementary Materials and Methods). Curiously, for some taxa with inversions confirmed by strong amplification of the Inv+ primer pairs, we also observed weak amplification in some of the Inv− primer pairs (**supplementary fig. S1**). We performed PCR on a taxon without any inversions (*A. ampullarius*) as well and saw no amplification or extremely weak amplification with the Inv+ primers. Additionally, multiple diffuse bands of PCR products with different sizes from the *trnT*-UGU/*accD* primer pair (Inv+) in *A. mollissimus* revealed that at least some copies of the plastome contain multiple copies of *trnT*-UGU. An alternate Ray (Boisvert et al. 2010) assembly of the *A. mollissimus* plastome using a different group of input reads included four copies of *trnT*-UGU as part of a 106-bp sequence repeated at least four times in tandem. This alternate assembly was scaffolded between the first occurrence of this tandem repeat and subsequent copies.

Supplementary Table S1

NCBI GenBank Accession Numbers for Annotated Plastomes and SRA Experiments for Read Data

Taxon	GenBank Accession No.	SRA Experiment
Astragalus acutirostris	MZ923736	SRX11969658
Astragalus agnicidus	MZ901206	SRX11815646
Astragalus americanus	MZ923737	SRX11969659
Astragalus ampullarioides	MZ923738	SRX11969670
Astragalus ampullarius	MZ923739	SRX11969673
Astragalus arrectus	MZ923740	SRX11969674
Astragalus bicristatus	MZ923741	SRX11969675
Astragalus bolanderi	MZ923742	SRX11969676
Astragalus calycosus var. *calycosus*	MZ923743	SRX11969677
Astragalus clevelandii	MZ923744	SRX11969678
Astragalus flexuosus var. *flexuosus*	MZ901207	SRX11815647
Astragalus gypsodes	MZ923745	SRX11969679
Astragalus lentiginosus var. *diphysus*	MZ923746	SRX11969660
Astragalus lentiginosus var. *mokiacensis*	MZ923747	SRX11969661
Astragalus malacus	MZ923748	SRX11969662
Astragalus mollissimus var. *mollissimus*	MZ901208	SRX11815648
Astragalus neglectus	MZ923749	SRX11969663
Astragalus nuttallianus var. *imperfectus*	MZ923750	SRX11969664
Astragalus obscurus	MZ923751	SRX11969665
Astragalus pattersonii	MZ923752	SRX11969666
Astragalus pectinatus	MZ923753	SRX11969667
Astragalus serenoi var. *serenoi*	MZ923754	SRX11969668
Astragalus tephrodes var. *chloridae*	MZ923755	SRX11969669
Astragalus toanus var. *toanus*	MZ923756	SRX11969671
Astragalus wootonii var. *wootonii*	MZ923757	SRX11969672

NOTE.—SRA read data submitted under BioProject numbers PRJNA755707 and PRJNA757771.

Supplementary Table S2

Genes Annotated in the Plastomes of 25 *Astragalus* Taxa by Category

Photosystem I (5)

psaA, *psaB*, *psaC*, *psaI*, *psaJ*

Photosystem II (15)

psbA, *psbB*, *psbC*, *psbD*, *psbE*, *psbF*, *psbH*, *psbI*, *psbJ*, *psbK*, *psbL*, *psbM*, *psbN*, *psbT*, *psbZ*

Cytochrome b/f complex (6)

petA, *petB**, *petD**, *petG*, *petL*, *petN*

ATP synthase (6)

atpA, *atpB*, *atpE*, *atpF**, *atpH*, *atpI*

NADH dehydrogenase (11)

*ndhA**, *ndhB**, *ndhC*, *ndhD*, *ndhE*, *ndhF*, *ndhG*, *ndhH*, *ndhI*, *ndhJ*, *ndhK*

Rubisco large subunit (1)

rbcL

RNA polymerase (4)

rpoA, *rpoB*, *rpoC1**, *rpoC2*

Ribosomal proteins (SSU) (11)

rps2, *rps3*, *rps4*, *rps7*, *rps8*, *rps11*, *rps12**, *rps14*, *rps15*, *rps18*, *rps19*

Ribosomal proteins (LSU) (8)

*rpl2**, *rpl14*, *rpl16**, *rpl20*, *rpl23*, *rpl32*, *rpl33*, *rpl36*

clpP, *matK* (2)

clpP[a], *matK*

Other genes (3)

accD, *ccsA*, *cemA*

Hypothetical chloroplast reading frames (*ycf*) (4)

ycf1, *ycf2*, *ycf3***, *ycf4*

Transfer RNAs (30)

trnA-UGC*, *trnC*-GCA, *trnD*-GUC, *trnE*-UUC, *trnF*-GAA, *trnfM*-CAU, *trnG*-GCC, *trnG*-UCC*, *trnH*-GUG, *trnI*-CAU, *trnI*-GAU*, *trnK*-UUU*, *trnL*-CAA, *trnL*-UAA*, *trnL*-UAG, *trnM*-CAU, *trnN*-GUU, *trnP*-UGG, *trnQ*-UUG, *trnR*-ACG, *trnR*-UCU, *trnS*-GCU, *trnS*-GGA, *trnS*-UGA, *trnT*-GGU, *trnT*-UGU, *trnV*-GAC, *trnV*-UAC*, *trnW*-CCA, *trnY*-GUA

Ribosomal RNAs (4)

rrn16, *rrn23*, *rrn4.5*, *rrn5*

NOTE.—Following the name of each category are the number of genes in parentheses; the number of introns in each gene is indicated by the number of asterisks.

[a] One intron in *clpP* was present in 18 taxa; no introns were present in *clpP* in seven taxa: *A. bolanderi*, *A. calycosus*, *A. malacus*, *A. neglectus*, *A. obscurus*, *A. pectinatus*, and *A. tephrodes*.

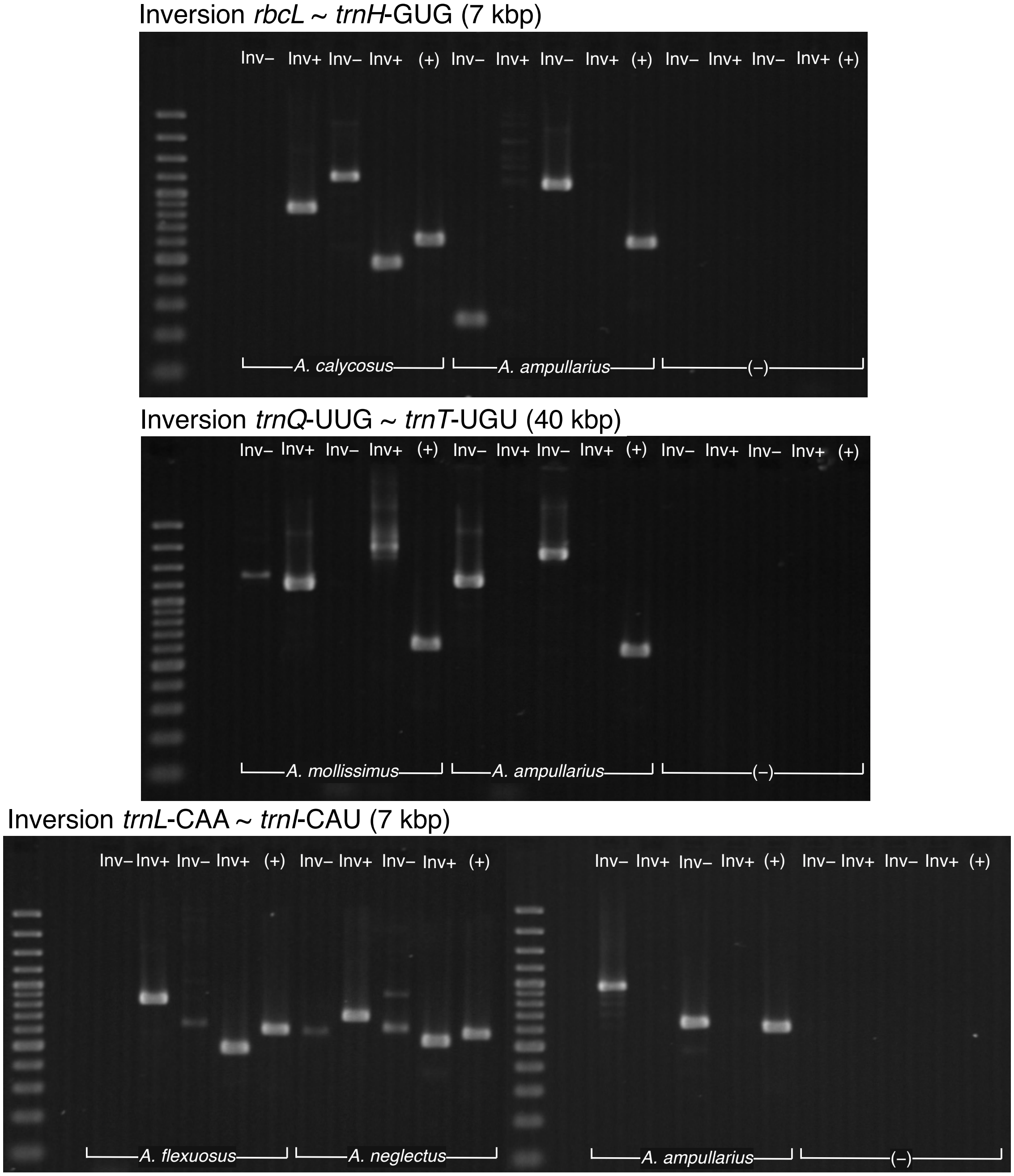

Supplementary Fig. S1.—PCR products from primers used to confirm the presence of three plastome inversions in four *Astragalus* species. In each case four, different combinations of four primers were used, two of which would be amplified if the inversion were present (Inv+) and two of which would be amplified if the inversion were not present (Inv−) (see **supplementary fig. S9**). For each inversion, PCR products from species with the inversion are shown along with one species with no inversions (*A. ampullarius*). (+) indicates a positive control for PCR (*trnL*-UAA intron). (−) indicates a negative control for PCR.

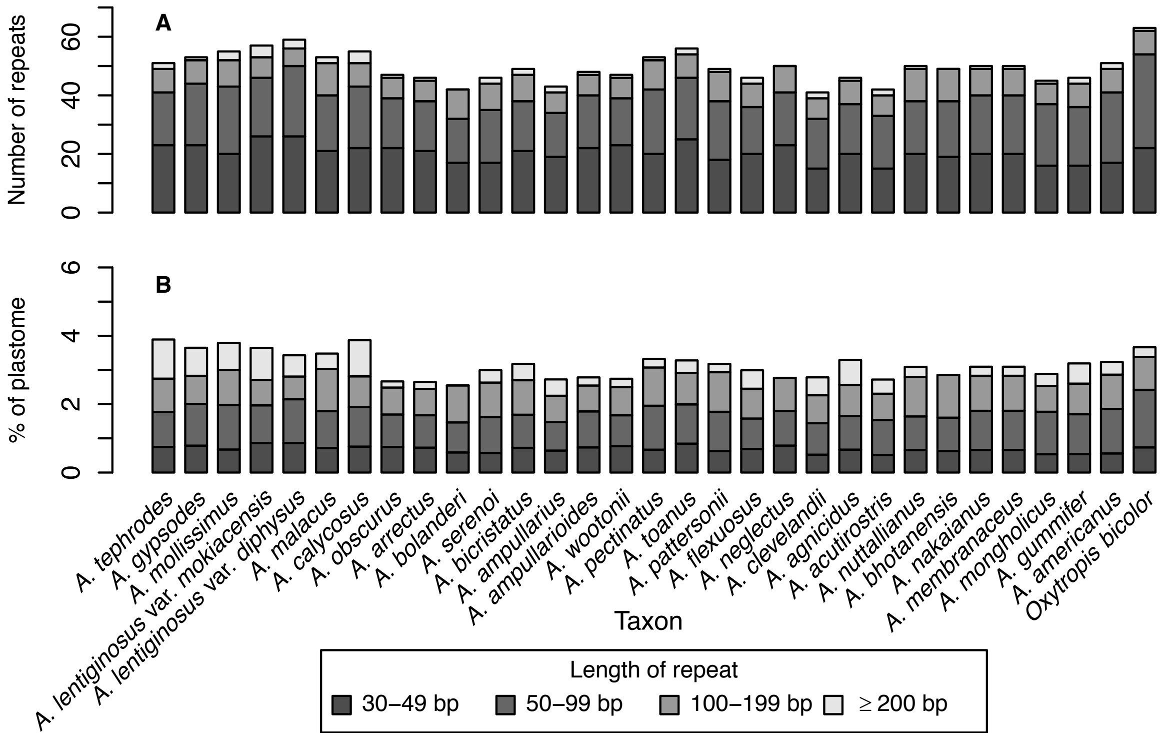

Supplementary Fig. S2.—Number of plastome repeats by length category (*A*) and percent of plastome in repeats by length category (*B*) for 30 *Astragalus* taxa and *Oxytropis bicolor*. Order of taxa from left to right is the same as in **figure 2** from top to bottom.

Supplementary Table S3

Phylogenetic Signal in Plastome Repeat Traits

Trait	Min.	Max.	Mean	SD	Pagel's λ	
Plastome length (bp)	121,590	124,016	122,924.9	581.7	0.990	$p = 0.129$
Total repeat length (bp)	3,109	4,776	3,866.1	477.4	0.947	$p = 0.044$*
Length repeats found in all taxa (bp)	1,332	2,208	1,582.2	207	0.000	$p = 1.000$
Length repeats found in majority of taxa (bp)	793	1,874	1,319.8	268.9	0.629	$p = 0.003$*
Length repeats found in minority of taxa (bp)	88	960	381.4	249.1	0.988	$p = 0.036$*
Length repeats unique to particular taxon (bp)	217	1,302	582.6	305.7	0.973	$p = 0.001$*
Total repeat content (%)	2.5479	3.8926	3.1449	0.3879	0.948	$p = 0.032$*
Frequency of repeats (repeats per 3 kbp)	1.0028	1.5433	1.2108	0.126	0.996	$p = 0.001$*
Repeats in 3 kbp windows variance to mean ratio	0.9024	2.3004	1.4	0.3	0.999	$p = 0.009$*

NOTE.—All traits were log-transformed before estimating phylogenetic signal, but the minimum, maximum, mean, and standard deviation reported for each trait are from the untransformed values; asterisks indicate λ is significantly > 0, indicating the presence of phylogenetic signal.

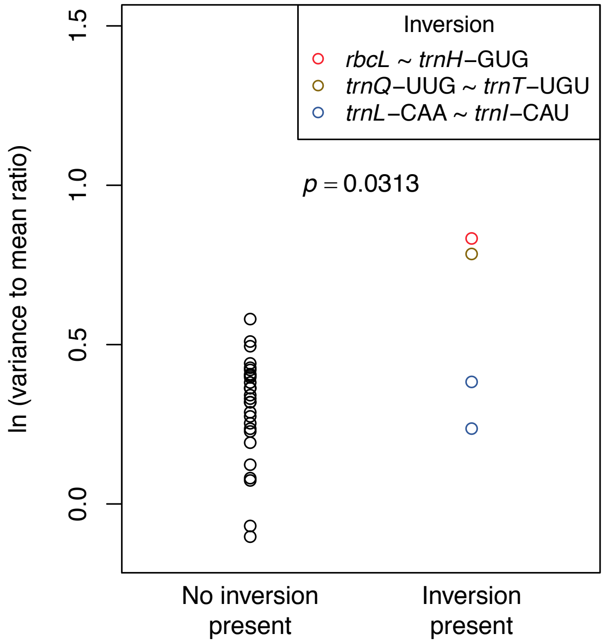

Supplementary Fig. S3.— Log-transformed ratio of variance to mean repeat count in 3-kbp windows in plastomes with and without inversions. Phylogenetic *t*-test performed with PGLS with Brownian motion–modeled covariance matrix in GLS framework with a continuous dummy variable with value 0 for plastomes without inversions and 1 for plastomes with inversions.

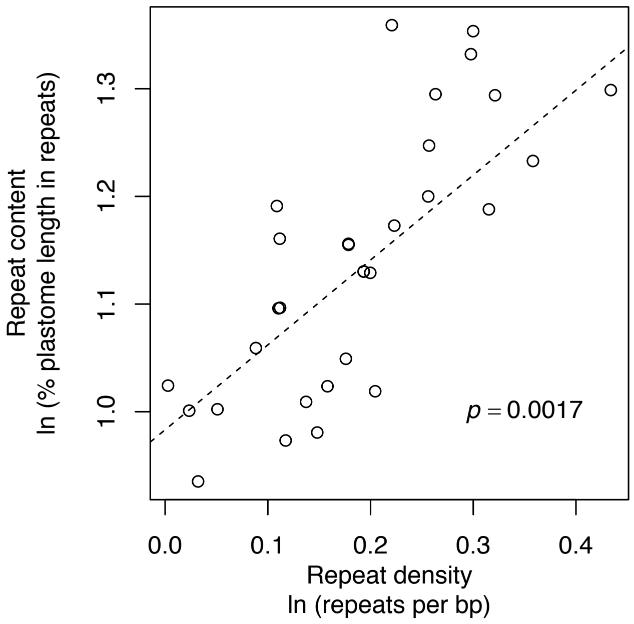

Supplementary Fig. S4.—Positive correlation between log-transformed repeat density and log-transformed repeat content. Regression performed using PGLS with Brownian motion–modeled covariance matrix and generalized least squares framework.

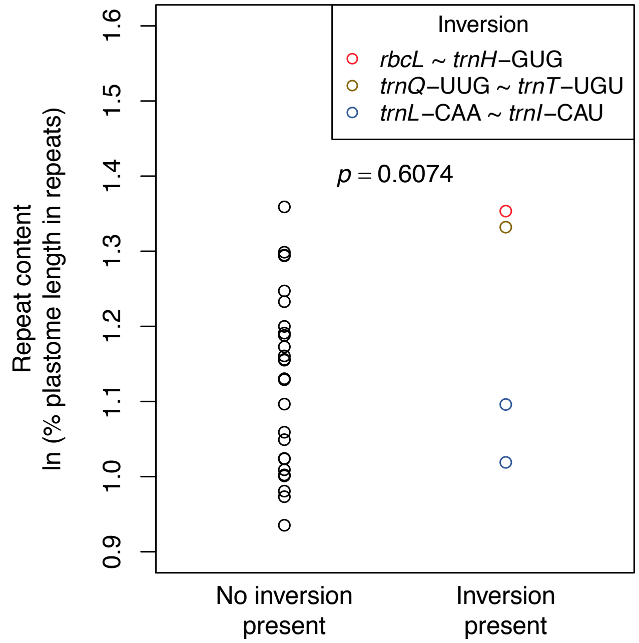

Supplementary Fig. S5.—Log-transformed repeat content in plastomes with and without inversions. Phylogenetic *t*-test performed with PGLS with Brownian motion–modeled covariance matrix in GLS framework with a continuous dummy variable with value 0 for plastomes without inversions and 1 for plastomes with inversions.

Supplementary Table S4

Observed and Expected Repeat Counts Within 1 kbp of Inversion Endpoint Locations in 30 *Astragalus* Taxa and *Oxytropis bicolor*

	Repeat count in non-endpoint regions		Repeat count in endpoint regions			
Taxon	Observed	Expected	Observed	Expected	χ^2 statistic	*p*-value
A. acutirostris	34	37.22	8	4.78	2.45	0.118
A. agnicidus	35	40.89	11	5.11	7.63	0.006*
A. americanus	43	45.31	8	5.69	1.06	0.304
A. ampullarioides	38	42.56	10	5.44	4.30	0.038*
A. ampullarius	34	38.27	9	4.73	4.32	0.038*
A. arrectus	37	40.82	9	5.18	3.18	0.075
A. bicristatus	39	43.52	10	5.48	4.21	0.040*
A. bolanderi	34	37.27	8	4.73	2.55	0.111
A. calycosus[a]	40	48.81	15	6.19	14.14	1.70×10^{-4}*
A. clevelandii	34	36.22	7	4.78	1.17	0.279
A. flexuosus[b]	37	40.86	9	5.14	3.26	0.071
A. gypsodes	43	47.00	10	6.00	3.01	0.083
A. lent. var. *diphysus*	43	52.28	16	6.72	14.47	1.42×10^{-4}*
A. lent. var. *mokiacensis*	43	50.40	14	6.60	9.37	0.002*
A. malacus	40	47.03	13	5.97	9.34	0.002*
A. mollissimus[c]	39	49.03	16	5.97	18.88	1.39×10^{-5}*
A. neglectus[b]	39	44.34	11	5.66	5.68	0.017*
A. nuttallianus	37	44.38	13	5.62	10.93	0.001*
A. obscurus	37	41.64	10	5.36	4.54	0.033*
A. pattersonii	38	43.55	11	5.45	6.35	0.012*
A. pectinatus	41	46.90	12	6.10	6.44	0.011*
A. serenoi	35	40.70	11	5.30	6.95	0.008*
A. tephrodes	37	45.16	14	5.84	12.86	3.36×10^{-4}*
A. toanus	42	49.68	14	6.32	10.53	0.001*
A. wootonii	38	41.73	9	5.27	2.97	0.085
A. bhotanensis	40	43.50	9	5.50	2.52	0.113
A. gummifer	39	40.81	7	5.19	0.71	0.399
A. membranaceus	40	44.35	10	5.65	3.76	0.052
A. mongholicus	37	39.90	8	5.10	1.87	0.172
A. nakaianus	40	44.34	10	5.66	3.76	0.053
Oxytropis bicolor	49	55.61	14	7.39	6.71	0.010*

NOTE.—Pearson χ^2 goodness-of-fit tests with 1 d.f. ($\alpha = 0.05$); asterisks indicate repeat counts significantly different from expectation given the length of endpoint regions.

[a] Inversion *rbcL* ~ *trnH*-GUG present

[b] Inversion *trnL*-CAA ~ *trnI*-CAU present

[c] Inversion *trnQ*-UUG ~ *trnT*-UGU present

Supplementary Table S5

Observed and Expected Repeat Counts Within Intergenic Spacers Containing Inversion Endpoint Locations in 30 *Astragalus* Taxa and *Oxytropis bicolor*

	Repeat count in non-endpoint spacers		Repeat count in endpoint spacers			
Taxon	Observed	Expected	Observed	Expected	χ^2 statistic	*p*-value
A. acutirostris	21	21.38	3	2.62	0.06	0.801
A. agnicidus	23	26.65	7	3.35	4.46	0.035*
A. americanus	29	29.38	4	3.62	0.04	0.834
A. ampullarioides	23	25.73	6	3.27	2.56	0.110
A. ampullarius	18	21.52	6	2.48	5.56	0.018*
A. arrectus	27	25.98	2	3.02	0.39	0.534
A. bicristatus	24	26.80	6	3.20	2.76	0.097
A. bolanderi	24	25.15	4	2.85	0.51	0.474
A. calycosus[a]	29	32.44	7	3.56	3.70	0.055
A. clevelandii	21	21.24	3	2.76	0.02	0.880
A. flexuosus[b]	20	22.13	5	2.87	1.79	0.182
A. gypsodes	27	27.40	4	3.60	0.05	0.821
A. lent. var. *diphysus*	30	34.59	9	4.41	5.39	0.020*
A. lent. var. *mokiacensis*	30	32.65	7	4.35	1.82	0.177
A. malacus	24	27.96	8	4.04	4.45	0.035*
A. mollissimus[c]	26	31.59	9	3.41	10.14	0.001*
A. neglectus[b]	25	27.60	6	3.40	2.24	0.135
A. nuttallianus	25	30.22	9	3.78	8.11	0.004*
A. obscurus	24	27.21	7	3.79	3.11	0.078
A. pattersonii	23	25.88	6	3.13	2.96	0.085
A. pectinatus	24	28.20	8	3.80	5.26	0.022*
A. serenoi	19	23.83	8	3.17	8.33	0.004*
A. tephrodes	25	29.19	8	3.81	5.19	0.023*
A. toanus	28	33.59	10	4.41	8.00	0.005*
A. wootonii	23	25.03	5	2.97	1.56	0.212
A. bhotanensis	30	31.02	5	3.98	0.30	0.586
A. gummifer	31	30.95	4	4.05	0.00	0.980
A. membranaceus	31	33.76	7	4.24	2.03	0.154
A. mongholicus	29	30.21	5	3.79	0.43	0.511
A. nakaianus	31	33.74	7	4.26	1.99	0.158
Oxytropis bicolor	35	40.78	11	5.22	7.23	0.007*

NOTE.—Pearson χ^2 goodness-of-fit tests with 1 d.f. ($\alpha = 0.05$); asterisks indicate repeat counts significantly different from expectation given the length of endpoint spacers.

[a] Inversion *rbcL* ~ *trnH*-GUG present

[b] Inversion *trnL*-CAA ~ *trnI*-CAU present

[c] Inversion *trnQ*-UUG ~ *trnT*-UGU present

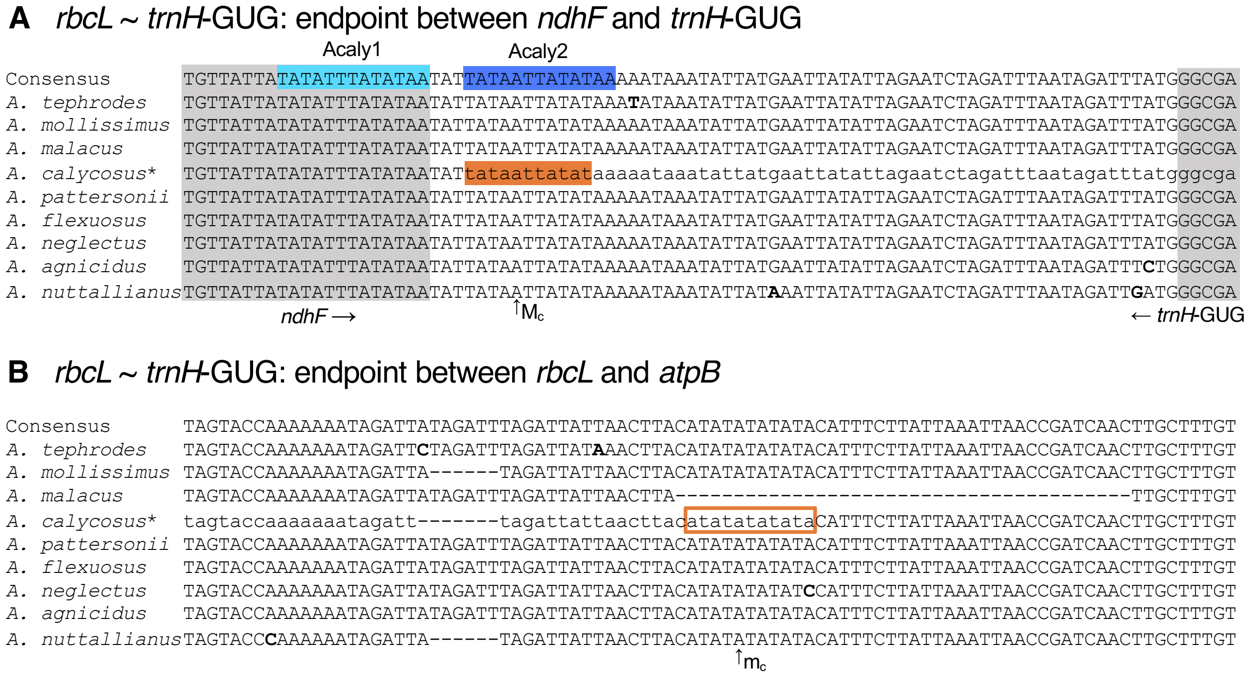

Supplementary Fig. S6.—Alignments of sequences found at endpoint locations of the *rbcL ~ trnH*-GUG inversion found in *Astragalus calycosus* (*). Lower-case nucleotides in *A. calycosus* are within the inversion and were reverted prior to alignment. At both inversion endpoints in *A. calycosus*, microhomologous sequences M_c (*A*) and m_c (*B*) pair at 9 of 11 sites. Repeat sequences Acaly1 and Acaly2 are separated by the *ndhF*/*trnH*-GUG inversion endpoint *(A)*. Majority consensus sequences are in the top row of each alignment. Bold nucleotides differ from the consensus.

A *trnQ*-UUG ~ *trnT*-UGU: endpoint between *trnL*-UAA and *trnT*-UGU

```
                                                Amoll1
Consensus      TATTCTTTTCAATTTGAAATCTAATCTACTATGACTAATATTAGTCATTATTTCAAAGATTGAAATAAATATTTGTATTTGAAATAGTAT
A. tephrodes   TATTCTTTTCAATTTGAAATCTAATCTACTATGACTAATATTAGTCATTATTTCAAAGATTGAAATAAATATTTGTATTTGAAATAGTAT
A. mollissimus*TATTCTTTTCAATTTGAAATCTAATCTACTATGACTAATATTAGTCATTATTTCAAAGATTGAAATAAATATTTATATTTGAAATAGTAT
A. malacus     TATTCTTTTCAATTTGAAATCTAATCTACTATGACTAATATTAGTCATTATTTCAAAGATTGAAATAAATATTTGTATTTGAAATAGTAT
A. calycosus   TATTCTTTTCAATTTGAAATCTAATCTACTATGACTAATATTAGTCATTATTTCAAAGATTGAAATAAATATTTGTATTTGAAATAGTAT
A. pattersonii TATTCTTTTCAATTTGAAATCTAATCTACTATGACTAATATTAGTCATTATTTCAAAGATTGAAATAAATATTTGTATTTGAAATAGTAT
A. flexuosus   TATTCTTTTCAATTTGAAATCTAATCTACTATGACTAATATTAGTCATTATTTCAAAGATTGAAATAAATATTTGTATTTGAAATAGTAT
A. neglectus   TATTCTTTTCAATTTGAAATCTAATCTACTATGACTAATATTAGTCATTATTTCAAAGATTGAAATAAATATTTGTATTTGAAATAGTAT
A. agnicidus   TATTCTTTTCAATTTGAAATCTAATCTACTATGACTAATATTAGTCATTATTTCAAAGATTGAAATAAATATTTGTATTTGAAATAGTAT
A. nuttallianusTATTCTTTTCAATTTGAAATCTAATCTACTATGACTAATATTAGTCATTATTTCAAAGATTGAAATAAATATTTGTATTTGAAATAGTAT

Consensus      TTTACATATCTATTATTTTTAGATTATATTTATATTATACATATTTAAAATAGAAGGTATTCTAAGTTCATTATAATATTAGAATATTTT
A. tephrodes   TTTACATATCTATTATTTTTAGATTATATTTA-----TACATATTTCAAATAGAAGGTATTCT-AGTTC----------TAGAAT-----
A. mollissimus*TTTACATATCTATTATTTTTAGATTATATTTAT----tacat--ttc-----gaa----ttctaagtccattataatattagaatatttt
A. malacus     TTTACATATCTATTATTTTTAGATTATATTTA-----TACATATTTCAAATAGAAGGTATTCT-AGTTC----------TAGAAT-----
A. calycosus   TTTACATATCTATTATTTTTAGATTATATTTATATTATACATATTTCAAATAGAAGGTATTCT-AGTTC----------TAGAAT-----
A. pattersonii TTTACATATCTATTATTTTTAGATTATATTTA-----TACATATTTAAAATAGAAGGTATTCT-AGTTC----------TAGAAT-----
A. flexuosus   TTTACATATCTATTATTTTTAGATTATATTTA-----TACATATTTAAAATAGAAGGTATTCT-AGTTC----------TAGAAT-----
A. neglectus   TTTACATATCTATTATTTTTAGATTATATTTA-----TACATATTTAAAATAGAAGGTATTCT-AGTTC----------TAGAAT-----
A. agnicidus   TTTACATATCTATTATTTTTAGATTATATTTA-----TACATATTTAAAATAGAAGGTATTCT-AGTTC----------TAGAAT-----
A. nuttallianusTTTACATATCTATTATATATAGATTATATTTA-----TACATATTTAAAAAAGAAGGTATTCT-AGTTC----------TAGAAT-----
                                                 ↑Mm                                Amoll2
Consensus      TTTCAATTTGTCAAGAGTCTTTTCAATTTTTCAATTTGTCAATAGTCTTTTCAATTTTTCAATTTGAAATCTAATCTACTATGACTAATA
A. tephrodes   -------------------------------------------------------------------------------------------
A. mollissimus*tttcaatttgtcaagagtcttttcaatttttcaatttgtcaatagtcttttcaatttttcaatttgaaatctaatctactatgactaata
A. malacus     -------------------------------------------------------------------------------------------
A. calycosus   -------------------------------------------------------------------------------------------
A. pattersonii -------------------------------------------------------------------------------------------
A. flexuosus   -------------------------------------------------------------------------------------------
A. neglectus   -------------------------------------------------------------------------------------------
A. agnicidus   -------------------------------------------------------------------------------------------
A. nuttallianus-------------------------------------------------------------------------------------------
                    Amoll2 cont.
Consensus      TTAGTCATTATTTCAAAGATTGAAATCTTATTATTATTATTAATTTAATTAATATATAATTTAATTAATAATTATTAATTTAATTAATAT
A. tephrodes   -------------------------TCTTATTAT----------------------TAATTTAATTAATTATTATTAATTTAATTAATAT
A. mollissimus*ttagtcattatttcaaagattgaaa------------------------------------------------------------------
A. malacus     -------------------------TCTTATTATTATTATTAATTTAATTAATATATAATTTAATTAATA--------------TAATAT
A. calycosus   -------------------------TCTTATTAT----------------------TAATTTAATTAATA-------------------T
A. pattersonii -------------------------TCTTATTAT----------------------TAATTTAATTAATA--------------------
A. flexuosus   -------------------------TCTTATTATTATTAT----------------TAATTTAATTAATA--------------TAATAT
A. neglectus   -------------------------TCTTATTAT----------------------TAATTTAATTAATA--------------------
A. agnicidus   -------------------------TCTTATTAT----------------------TAATTTAATTAATA--------------TAATAT
A. nuttallianus-------------------------TCTTATTAT----------------------TAATTTAATTAATA--------------------

Consensus      ATAATAATATATTATAGAAAAAATTGATTATTAAATAATAGATCATAAAATAGAAGAGATATAATAAAAATAAATAAGAATTATGTGTTA
A. tephrodes   ATTATTATATATTATAGAAAAAATTGATTATTAAATAATAGATCATAAAATAGAAGAGATATAATAAAAATAAATAAGAATTATGTGTTA
A. mollissimus*-------------------------------------------------------------------------------------------
A. malacus     ATAATAATATATTATAGAAAAAATTGATTATTAAATAATAGATCATAAAATAGAAGAGATAT------AATAAATAAGAATTATGTGTTA
A. calycosus   ATAATAATATATTATAGAAAAAATTGATTATTAAATAATAGATCATAAAATAGAAGAGATAT------AATAAATAAGAATTATGTGTTA
A. pattersonii ATAATAATATATTATAGAAAAAATTGATTATTAAATAATAGATCATAAAATAGAAGAGATAT------AATAAATAAGAATTATGTGTTA
A. flexuosus   ATAATAATATATTATAGAAAAAATTGATTATTAAATAATAGATCATAAAATAGAAGAGATAT------AATAAATAAGAATTATGTGTTA
A. neglectus   -------------------------------------------------------------------------------------------
A. agnicidus   ATAATAATATATTATAGAAAAAATTGATTATTAAATAATAGATCATAAAAGAGAAGAGATAT------AATAAATAAGAATTATGTGTTA
A. nuttallianus----TAATATATTATAGAAAAAATTGATTATTAAATAATAGCTCATAAAATAGAAGAGATAT------AATAAATAAGAATTATGTGTTA
                Amoll2 cont.
Consensus      GATGTAAATATTTGATTTATTATTTAATATATATATTTAATATATATATTATAAATATTTC
A. tephrodes   GATGTAAATATTTGATTTATTATTTAATATATATATTTAATATATATATTATAAATATTTC
A. mollissimus*----taaatatttgatttat-------------tatttaatatatatattataaaatttc
A. malacus     GATGTAAATATTTGATTTAT-------------TATTTAATATATATATTATAAATATTTC
A. calycosus   GATGTAAATATTTGATTTAT-------------TATTTAATATATATATTATAAATATTTC
A. pattersonii GATGTAAATATTTGATTTAT-------------TATTTAATATATATATTATAAATATTTC
A. flexuosus   GATGTAAATATTTGATTTAT-------------TATTTAATATATATATTATAAATATTTC
A. neglectus   ---------------------------------------TAATATATATATTATAAATATTTC
A. agnicidus   GATGTAAATATTTGATTTAT-------------TATTTAATATATATATTATAAATATTTC
A. nuttallianusGATGTAAATATTTGATTTCT-------------TATTTCATATATATATTCTAAATATTTC
```

Supplementary Fig. S7 (continued on next page).—Alignments of sequences found at endpoint locations of the *trnQ*-UUG ~ *trnT*-UGU inversion found in *Astragalus mollissimus* (*).

B *trnQ*-UUG ~ *trnT*-UGU: endpoint between *trnQ*-UUG and *accD*

Consensus	ATGTAGATGTAGATTCATAAGCGACTCGATTTTTTTTGTCTTTTTTTATTCAAATCTTATCTTAGTTTTTATATTATTATTTATTATATTCT
A. tephrodes	ATGTAGATGTAGATTCATAAGCGACTCGATTTTTTTTGTCTTTTTTTATTCAAATCTTATCTTAGTTTTTAT**T**TTATTAT-----------T
*A. mollissimus**	atgtagatgtagattcataag**a**gactcgattttttttgtctttttttattcaaatcttatcttagtttttatattattat-----------t
A. malacus	ATGTAGATGTAGATTCATAAGCGACTCGATTTTTTTTGTCTTTTTTTATTCAAATCTTATCTTAGTTTTTATATTATTATTT**T**TTATATT**A**T
A. calycosus	ATGTAGATGTAGATTCATAAGCGACTCGATTTTTTTT**T**TCTTTTTTTATTCAAATCTTATCTTAGTTTTTATATTATTAT------------
A. pattersonii	ATGTAGATGTAGATTCATAAGCGACTCGATTTTTTTTGTCTTTTTTTATTCAAATCTTATCTTAGTTTTTATATTATTATTTATTATATTCT
A. flexuosus	ATGTAGATGTAGATTCATAAGCGACTCGATTTTTTTTGTCTTTTTTTATTCAAATCTTATCTTAGTTTTTATATTATTATTTATTATATTCT
A. neglectus	ATGTAGATGTAGATTCATAAGCGACTCGATTTTTTTTGTCTTTTTTTATTCAAATCTTATCTTAGTTTTTATATTATTAT-----------T
A. agnicidus	ATGTAGATGTAGATTCATAAGCGACTCGATTTTTTTTGTCTTTTTTTATTCAAATCTTATCTTA**A**TTTTTATATTATTAT-----------T
A. nuttallianus	ATGTAGATGTAGATTCATAAGCGACTCGATTTTTTTTGTCTTTTTTTATTCAAATCTTATCTTAGTTTTTATATTATTAT-----------T

Consensus	TTTTATATTATTATTATTATTATATTGTAATAATTGTGGATAATAGTTTAAATATTAAATATATTGATTGAATAAATGACTACGCACAAT
A. tephrodes	TTTTATATTATTATTA-------ATTGTAATAATTGTGGATAATAGTTT**C**AATAT**G**AAATATATTGATTGAATAAATGACTACGCACAAT
*A. mollissimus**	ttttatattattattattattatattgta----TTGTGGATAA---
A. malacus	TTTTATATTATTATTA------TATTGTAATAATTGTGGATAATAGTTTAAATA------------------------------------
A. calycosus	--------------TA------TATTGTAATAATTGTGGATAATAGTTT**C**AATATTAAATATATTGATTGAATAAATGACTACGCACAAT
A. pattersonii	T**A**TTATATTATTATTA------TATTGTAATAATTGTGGATAATAGTTTAAATATTAAATATATTGATTGAATAAATGACTACGCACAAT
A. flexuosus	T**A**TTATATT**C**TTATTA------TATTGTAATAATTGTGGATAATAGTTTAAATATTAAATATATTGATTGAATAAATGACTACGCACAAT
A. neglectus	TTTTATATTATTATTA------TATTGTAATAATTGTGGATAATAGTTTAAATATTAAATATATTGATTGAATAAATGACTACGCACAAT
A. agnicidus	TTTTATATTATTATTA------TATTGTAATAATTGTGGATAATAGTTTAAATATTAAATATATTGATTGAATAAATGACTACGCACAAT
A. nuttallianus	TTTTATATTATTATTA------TATTGTAATAATTGTGGATAATAGTTTAAATATTAAATATATTGATTGAATAAATGACTACGCACAAT

↑m_m

Consensus	TTCAGAATCCATTAAATACCAGATCTAACTAAAAAGAATTTTATGGTAAAACAAAACATCGTTTTAAATGATGTGCTAAAAAAATACA
A. tephrodes	TTCAGAATCCATTAAATACCAGATCTAACTAAAAAGAATTTTATGGT-----AAAACATCGTTTTAAATGATGTGCTAAAAAAATACA
*A. mollissimus**	--TCGTTTTAAATGATGTGCTAAAAAAATACA
A. malacus	--
A. calycosus	TTCAGAATCCATT**T**A---
A. pattersonii	TTCAGAATCCATTAAATACCAGATCTAACTAAAAAGAATTTTATGGT-----AAAACATCGTTTTAAATGATGT**A**CTAAAAAAATACA
A. flexuosus	TTCAGAATCCATTAAATACCAGATCTAACTAAAAAGAATTTTATGGT-----AAAACATCGTTTTAAATGATGT**A**CTAAAAAAATACA
A. neglectus	TTCAGAATCCATTAAATACCAGATCTAACTAAAAAGAATTTTATGGTAAAACAAAACATCGTTTTAAATGATGT**A**CTAAAAAAATACA
A. agnicidus	TTCAGAATCCATTAAATACCAGATCTAACTAAAAAGAATTTTATGGT-----AAAACATCGTTTTAAATGATGTGCTAAAAAA**G**TACA
A. nuttallianus	TTCAGAATCCATTAAATACCAGATCTAACTAAAAAGAATTTTATGGT-----AAAACATCGTTTTAAATGATGTGCTAAAAAA**G**TACA

Supplementary Fig. S7 (continued).—Alignments of sequences found at endpoint locations of the *trnQ*-UUG ~ *trnT*-UGU inversion found in *Astragalus mollissimus* (*). Lower-case nucleotides in *A. mollissimus* are within the inversion and were reverted prior to alignment. At both inversion endpoints in *A. mollissimus*, microhomologous sequences M_m (*A*) and m_m (*B*) pair at 5 of 6 sites (9 of 10 sites if four bases missing from *A. mollissimus* just past the *trnQ*-UUG/*accD* endpoint are included). Repeat sequences Amoll1 and Amoll2 are separated by the *trnL*-UAA/*trnT*-UGU inversion endpoint (*A*). Majority consensus sequences in are the top row of each alignment. Bold nucleotides differ from the consensus.

A *trnL*-CAA ~ *trnI*-CAU: endpoint between *rpl23* and *trnI*-CAU

```
                                  Aflex1                  Anegl1                              Anegl1 cont.
Consensus         AATTATTTAATAAATAATAATTAAAAAATAAAATAATAATCTAATTGAAGTTTAGTAATTAGTAATAATAATAGTTTAGTTTTAGTAATA
A. malacus        AATAATTTAATAA-TAATAATTAAAAAATAAAATAATAATCTAATTCAAG------------------------------TTTAG-----
A. pattersonii    AATTATTT----------------------AAATAATAATCTAATTGAAG------------------------------TTTAGTAAT-
A. flexuosus*     AATTATTTAATAAATAATAATTAAAAAATAAAATAATAATCTAATTGAAG----------------------------------------
A. neglectus*     AATTATTTAAT-----------------TTAAATAATAATCTAATTGAAG------------------------------TTTAGTAAT-
A. agnicidus      AATAATTTAATAA-TAATAATTAAAAAATAAAATAATAATCTAATTGAAG----------------------------------------
A. nuttallianus   AATTACTT----------------------AAATAATAATCGAATTGAAGTTTAGTAATTAGTAATAATAATAGTTTAGTTTTAGTAATA

Consensus         ATAATAGTTTAGTTTTAGTAATTARTAATAATTGAAGTWTAGTAAGAAAATAATTTATTGAAATTATTTAATAAATAATGTAATAATAAT
A. malacus        -------------------------------TTGAAGTTTAG--------------------------------------------CAAT
A. pattersonii    ---------------TAGTAA-TAATAATAATTGAAGTAAAGTAAGAAAATAATTTATTGAAATTATTTAATAAATAATGTAATAATAAT
A. flexuosus*     ------------------------------------------------------------------------------------------
A. neglectus*     ------------------------------------------TAAAGTAATAA----------AATAATAAATTGAAATTTATT--GAAA
A. agnicidus      ------------------------------------------------------------------------------------------
A. nuttallianus   ATAATAGTTTAGTTTTAGTAATTAGTAATA-TT------TAG--------------------------------------TAATAATAAT

                                                                                                      Aflex2
Consensus         AATTGAAGTAAAGTAAGAAAATAATTTATTGAAATTATTTAATAAAGTAAKAACTCTATAAATAATAATAAATTGAAATTTATTATTAAA
A. malacus        AATTGAAG----------------------------------TAAAATAATAA-----------ATAATAAATTGAAATTTATT--TAAA
A. pattersonii    AATTGAAGTAAAGTAAGAAAATAATTTATTGAAATTATTTAATAAAGTAAGAA----------AATAATTTATTGAAA------------
A. flexuosus*     ------------------------------------------TAAAGTAATAActctataaatctctataaattgaaatttattatttta
A. neglectus*     ------------------------------------------TAAAGTAATAA----------AATAATAAATTGAAATTTATT--GAAA
A. agnicidus      --------------------------------------------------TAA----------AATAATAAATTGAAA------------
A. nuttallianus   AATTGAAG----------------------------------TAAAGTAATAA--------------AT---TTCAA-TTTATT--TAAA
                      Aflex2 cont.               Aflex2 cont.      Anegl2  ↑Mf                       Anegl3
Consensus         TTATTTAATAAATAATTTAWTAATAATAATTAAAAAATAAAATAATAATCTAATTGAAGTWWAGTCTTGTTTTCAAATAATAATCTAATT
A. malacus        TTATTTAATAAATAATTTAATAATAATAATTAAAAAATAAAATAATAATCTAATT-----------------------------------
A. pattersonii    TTATTTAATAAATAATGTATTAATAATAATTAAAAAATAAAATAATAATCTAATTGAAGTAAAGTCTTGTTTTCAAATAATAATCTAATT
A. flexuosus*     ttatttaataaataa-----------taattaaataataa-----taatctaatt-----------------------------------
A. neglectus*     TTATTTAATAAATAATTTATTAATAATAATTAAAAAATAaaataataatctaattgaagtttagtctt-------aataataatctaatt
A. agnicidus      TTATTTAATAAATAATTTAATAATAATAATTAAAAAATAAAATAATAATCTAATT-----------------------------------
A. nuttallianus   TTATTTAATAAATAA------------------------------TAATCTAAT------------------------AATAATCTAATT
                                                           ↑Mn
                         Anegl3 cont.
Consensus         GATAATCTAATTGAAGTTTAGTCTTGTTTT
A. malacus        ------------CAAGTTTAGTCTTGTTTT
A. pattersonii    ------------GAAGTTTAGTCTTGTTTT
A. flexuosus*     ------------gaagtttagtcttgtttt
A. neglectus*     ------------gaagtttagtcttgtttt
A. agnicidus      ------------GAAGTTTAGTCTTGTTTT
A. nuttallianus   GATAATCTAATTGAAGTTTAGTCTTGTTTT
```

Supplementary Fig. S8 (continued on next page).—Alignments of sequences found at endpoint locations of the *trnL*-CAA ~ *trnI*-CAU inversion found in *Astragalus flexuosus* and *A. neglectus*(*).

B *trnL*-CAA ~ *trnI*-CAU: endpoint between *trnL*-CAA and *ndhB*

```
Consensus      GTCGGACATCCAATTGGTTCGATTTGAATTATCCGAAAGATAGAGATMTTATATATTTATATTATTMTATTATATTCGATAAAAAATGGA
A. malacus     GTCGGACATCCAATTGGTTCGATTTGAATTATCCGAAAGATAGAGATATTATATATTTATATTATTATATTATATTCGATAAAAAATGGA
A. pattersonii GTCGGACATCCAATTGGTTCGATTTGAATTATCCGAAAGATAGAGATATTATATATTTATATTATTATATTATATTCGATAAAAAATGGA
A. flexuosus*  gtcggacatccaattggttcgatttgaattattcgaaagatagagatattatatatttatattat-----tatattcgataaaaaatgga
A. neglectus*  gtcggacatccaattggttcgatttgaattatccgaaagatagagatattatatatttATATTATTCTATTATATTCGATAAAAAACGGA
A. agnicidus   GTCGGACATCCAATTGGTTCGATTTGAATTATCCGAAAGATAGAGATCTTATATATTTATATTATTCTATTATATTCGATAAAAAATGGA
A. nuttallianusGTCGAACATCCAATTGGTTCGATTTAAATTATCCGAAAGATAGAGATATTATATATTTATATTAT-----TATATTCGATAAAAAATGGA
                                                                ↑mn

Consensus      CAATCAAACCTATTTTTGGCTTCAATAGAAAGAAGCCAAAAGAGGTAATATAGGGTCTTAAATAATGAGAGATATTTAAAATTAAAAAGC
A. malacus     CAATCAAACCTATTTTTGGCTTCAATAGAAAGAAGCCAAAAGAGGTAATATAGGGTCTTAAATAATGAGAGATATTTAAAATTAAAAAGC
A. pattersonii CAATCAAACCTATTTTTGGCTTCAATAGAAAGAAGCCAAAAGAGGTAATATAGGGTCTTAAATAATGAGAGATATTTAAAATTAAAAAGC
A. flexuosus*  caatcaaacctatttttggcttcaatagaaagaagccaaaagaggtaatatagggtcttaaataatgagagATATTTAAAATTAAAAAGC
A. neglectus*  CAATCAAACCTATTTTTGGCTTCAATAGAAAGAAGCCAAAAGAGGTAATATAGGGTCTTAAATAATGAGAGATATTTCAAATTAAAAAGC
A. agnicidus   CAATCAAACCTATTTTTGGCTTCAATAGAAAGAAGCCAAAAGAGGTAATATAGGGTCTGAAATAATGAGAGATATTTAAAATTAAAAAGC
A. nuttallianusCAATCAAACCTATTTTTGGCTTCAATAGAAAGAAGCCAAAAGAGGTAATATAGGGTCTTAAATAATGAGAGATATTTAAAATTAAAAAGC
                                                                              ↑mf
```

Supplementary Fig. S8 (continued).—Alignments of sequences found at endpoint locations of the *trnL*-CAA ~ *trnI*-CAU inversion found in *Astragalus flexuosus* and *A. neglectus* (*). Lower-case nucleotides in *A. flexuosus* and *A. neglectus* are within their respective inversions and were reverted prior to alignment. At both inversion endpoints in *A. flexuosus* microhomologous sequences M_f (*A*) and m_f (*B*) pair at the outermost four sites (8 of 10 sites if one base removed from the *trnL*-CAA/*ndhB* endpoint sequence). Repeat sequences Aflex1 and Aflex2 are separated by the *rpl23*/*trnI*-CAU inversion endpoint in *A. flexuosus* (*A*). At both inversion endpoints in *A. neglectus*, microhomologous sequences M_n (*A*) and m_n (*B*) pair at the five outermost sites (11 of 12 sites if one base removed from the *trnL*-CAA/*ndhB* sequence). Repeat sequences Anegl1 and Anegl2/Anegl3 are separated by the *rpl23*/*trnI*-CAU inversion endpoint in *A. neglectus* (*A*). Majority consensus sequences are in the top row of each alignment. Bold nucleotides differ from the consensus.

Supplementary Materials and Methods

Sampling, DNA Extraction, and Sequencing

Before library preparations using the NEBNext Ultra II DNA Kit (New England Biolabs, Ipswich, MA), concentrations of whole genomic DNAs were measured using a Qubit dsDNA High Sensitivity Assay Kit with a Qubit 4 Fluorometer (Invitrogen, Waltham, MA). Up to 250 ng of purified whole genomic DNA were used as input when enough DNA was available, but libraries were successfully prepared from as little as 55 ng. DNAs were fragmented with a Bioruptor Pico sonicator (Diagenode, Denville, NJ) at OSU CGRB to peak a fragment size of either 300 bp or 400 bp. Some DNAs that were fragmented to begin were sonicated with a shortened protocol or not at all.

All NEBNext libraries had fragment size distribution estimated on 2% agarose gels, and final library concentration was again determined using a Qubit fluorometer as described above. Libraries were prepared for 71 additional samples not included here, and all 96 libraries were combined into eight pools approximately equimolarly by adding equivalent masses of DNA from libraries with similar insert sizes. The size distribution and concentration of pools were quantified with an Agilent 4200 TapeStation D5000 ScreenTape (Santa Clara, CA) and with qPCR on an Applied Biosystems QuantStudio 5 Real-Time PCR System (Waltham, MA) and mixed equimolarly at the ASU Genomics Facility.

Sequence Data Preprocessing and Plastome Assembly

Illumina HiSeq reads from the three initial samples had adapters trimmed and were quality trimmed using Trimmomatic v. 0.33 (Bolger et al. 2014) using the settings `ILLUMINACLIP:TruSeq3-PE.fa:2:30:10` and quality trimming with settings `LEADING:3`

`TRAILING:3 SLIDINGWINDOW:4:15` and a minimum read length of 36 bp. We used Ray v. 2.3.1 (Boisvert et al. 2010) with *k*-mer length of 31 to perform *de novo* assembly on several groups 6–12 million read pairs for each of the three initial samples. Single contigs or scaffolds with the complete plastid genome were assembled from multiple read groups for each sample, and we used the longest assembly that consisted of a single contig for each sample going forward.

NextSeq reads that had been adapter-trimmed and filtered for contamination were mapped to the three HiSeq-sequenced reference plastomes we had assembled plus three NCBI RefSeq plastomes from *Astragalus mongholicus* Bunge (NC_029828), *A. nakaianus* Y.N. Lee (NC_028171), and *Oxytropis bicolor* Bunge (NC_047482). These reads mapping to reference plastomes were then assembled using the `tadpole.sh` assembler of BBMap (Bushnell B, https://sourceforge.net/projects/bbmap, last accessed July 13, 2018) to create reference contigs for each sample. We then mapped the contamination-filtered reads a second time to the sample reference contigs to get a final pool of plastome reads, which we then merged overlapping pairs of with `bbmerge.sh` (Bushnell et al. 2017).

Plastome read normalization to approximately 100x coverage was performed prior to assembly with `bbnorm.sh` of the BBMap (Bushnell B, https://sourceforge.net/projects/bbmap, last accessed July 13, 2018) package. SPAdes plastome assemblies included error correction with BayesHammer (Nikolenko et al. 2013) and post-processing mismatch correction (`-careful` option of SPAdes) with BWA (Li and Durbin 2010), with automatically selected *k*-mer lengths of 21, 33, and 55 nt.

Plastome Annotation

For GeSeq (Tillich et al. 2017) annotations of our three HiSeq-sequenced plastomes, in addition to the default GeSeq settings (https://chlorobox.mpimp-golm.mpg.de/geseq.html, last accessed July 5, 2020), we also included annotation of *trans*-spliced *rps12*, HMMer (Wheeler and Eddy 2013) HMM search, both ARAGORN (Laslett and Canback 2004) and tRNAscan-SE (Lowe and Eddy 1997) tRNA searches, and MPI-MP chloroplast references (Tillich et al. 2017). Annotations using PGA (Qu et al. 2019) and GeSeq of our three reference plastomes were checked manually against the annotated plastomes of *A. mongholicus* (NC_029828), *A. nakaianus* (NC_028171), *Oxytropis bicolor* (NC_047482), *Cicer arietinum* L. (NC_011163), *Glycine max* (L.) Merr. (NC_007942), and *Lotus japonicus* (Regel) K. Larsen (NC_002694) as well as non-legumes *Amborella trichopoda* Baill. (NC_005086), *Arabidopsis thaliana* (L.) Heynh. (NC_000932*), Nicotiana tabacum* L. (NC_001879), and *Prunus persica* (L.) Batsch (NC_014697).

Plastome Phylogeny Estimation

IQ-TREE (Minh et al. 2020) ML tree search was performed along with 1,000 ultrafast bootstrap replicates (Hoang et al. 2017). The ModelFinder (Kalyaanamoorthy et al. 2017) option of IQ-TREE selected the TVM+F+R2 model (transversion model with empirical base frequencies and two FreeRate categories) as the best model of sequence evolution using the Bayesian Information Criterion (BIC). The extended majority-rule consensus tree with optimized branch lengths, which was used for subsequent analyses, had the same topology as the ML tree. *Oxytropis bicolor* was specified as the outgroup for IQ-TREE and the tree was rooted to the

midpoint of the branch leading to *Oxytropis bicolor* using ape v. 5.4 (Paradis and Schliep 2019) and phytools v.0.7-47 (Revell 2012) in R (R Core Team 2020) prior to analyses.

Identification and Confirmation of Inversions

Four primers were designed to confirm the presence (or absence) of each plastome inversion (**supplementary fig. S9**). with two primer pair combinations that should amplify if the inversion is present (Inv+) and two that should amplify if the inversion is absent (Inv−), similar to the strategies of Kim et al. (2005) and Lee et al. (2007). Each primer was designed in a locus adjacent to one of the endpoints with the 3' end of the primer closest to the inversion endpoint. Primers were designed using Primer3 v. 2.3.4 (Untergasser et al. 2012) within Geneious v. 9.1.8 (Kearse et al. 2012) using sequences from one taxon with each of the inversions detected (*Astragalus calycosus*, *A. mollissimus*, and *A. flexuosus* for the *rbcL* ~ *trnH*-GUG, *trnQ*-UUG ~ *trnT*-UGU, and *trnL*-CAA ~ *trnI*-CAU inversions, respectively). Primer names and sequences are in **supplementary table S7**.

We performed PCR on all taxa with apparent plastome inversions using all four primer combinations for the inversion detected. We also did PCR on another taxon without any apparent inversions (*A. ampullarius*) for each set of primers to confirm amplification would work with the Inv− primer combinations when each inversion was absent. A positive control was used for each sample with primers to amplify the *trnL*-UAA intron (based on those of Taberlet et al. [2007]), and a negative control was also run for each PCR setup using nuclease-free water. PCR reactions contained a final concentration of 1× One*Taq* Standard Reaction Buffer, 200 μM of each dNTP, and 0.2 μM of each primer as well as 0.625 units of One*Taq* DNA polymerase (New England Biolabs, Ipswich, MA) and 2 ng of template DNA in a total volume of 25 μL. After an initial

denaturation at 94 ºC for 30 s, 30 PCR cycles of denaturation at 94 ºC for 30 s, annealing at a specific temperature for each primer set (**supplementary table S8**) for 30 s and extension at 68 ºC for a specific time for each primer set (**supplementary table S8**) were performed followed by a final extension at 68 ºC for 5 min.

PCR products were visualized on 2% agarose gels, and PCR products from Inv+ primer pairs were Sanger sequenced by Eton Biosciences (San Diego, CA). Base calls from sequence chromatograms were edited using Geneious.

Repeat Identification and Distribution

To identify plastome repeats, we used `blastn` (`-task blastn`) with a word size of seven and maximum *E*-value of 1×10^{-6} to BLAST (Altschul et al. 1990) each plastome against itself and then categorized hits as self-complements, dispersed direct repeats, or dispersed inverted repeats. Tandem repeats were identified with command-line TRF (Benson 1999) using the default settings of the web service (`2 7 7 80 10 50 500`; https://tandem.bu.edu/trf/trf.html, last accessed June 17, 2020).

In classifying repeat groups using Markov clustering, BLAST hits were obtained using the same settings as those used to identify the repeats within each plastome. The negative natural log of the *E*-value for hits were used as edge weights for clustering using MCL(Van Dongen 2002) with an inflation value (`-I`) of 1.4.

The repeat count and content within 1 kbp of each inversion endpoint feature (identified with MUMmer) were tabulated using BEDtools (Quinlan and Hall 2010) `slop` to increase the size of each feature by 1 kbp in both directions, and then `intersect -c` and `intersect -wao`

for counts and content, respectively. Intergenic spacer intervals were extracted using BEDtools `complement`, and repeats in spacers were counted with `intersect -c`.

Comparative Method Tests of Repeat Distribution and Inversion Status

We estimated phylogenetic signal in a number of plastome characters with the extended majority-rule consensus phylogeny (which had a topology identical to the ML tree) and the `phylosig` function of the phytools (Revell 2012) package of R (R Core Team 2020) using Pagel's (1999) λ, testing the hypothesis that the estimated value of λ differed from zero (no phylogenetic signal). We used phylogenetic least squares regression (PGLS) to determine if there was a correlation between repeat content and the density of repeats (count) using Brownian motion models of trait evolution with the ape (Paradis and Schliep 2019) and nlme (Pinheiro et al. 2020) packages of R (R Core Team 2020). Phylogenetic *t*-tests were performed by encoding inversion status as a continuous dummy variable and performing PGLS as above. The *p*-value associated with the estimated slope is for a likelihood ratio test between models in which the slope is fixed at zero and the estimated slope.

Supplementary Table S6

Collections and Taxonomic Information for 25 Astragalus Taxa with Assembled Plastomes

Taxon	Section	Isolate	Voucher specimen	Herbarium	Locality	Date collected
A. acutirostris S. Watson	*Leptocarpi*	JA 16753	J. André 16753[a]	RSA	USA, CA, San Bernardino Co.	28 Mar 2011
A. agnicidus Barneby	*Miselli*	JB s.n.	J. Brown s.n.[a]	OSC	USA, CA, Mendocino Co.	20 Jun 2011
A. americanus (Hook.) M.E. Jones	*Phaca*	JLMC 8296	J.L.M. Charboneau 8296[a]	RM	USA, MT, Phillips Co.	23 Jul 2011
A. ampullarioides (S.L. Welsh) S.L. Welsh	*Ampullariii*	JLMC 9864-3	J.L.M. Charboneau 9864	ZNP	USA, UT, Washington Co.	17 May 2017
A. ampullarius S. Watson	*Ampullariii*	JLMC 9863-1	J.L.M. Charboneau 9863	ARIZ	USA, UT, Kane Co.	16 May 2017
A. arrectus A. Gray	*Reventi-Arrecti*	LH 2931	L. Hufford 2931[a]	RSA	USA, WA, Whitman Co.	28 May 1998
A. bicristatus A. Gray	*Bicristati*	DSB 2722	D.S. Bell 2722[a]	RSA	USA, CA, San Bernardino Co.	30 Jun 2011
A. bolanderi A. Gray	*Hesperonix*	GKH 16809	G. Helmkamp 16809[a]	ARIZ	USA, CA, El Dorado Co.	20 Aug 2010
A. calycosus Torr. ex S. Watson var. *calycosus*	*Scaposi*	JLMC 9827	J.L.M. Charboneau 9827	ARIZ	USA, NV, Elko Co.	17 Jun 2016
A. clevelandii Greene	*Micranthi*	JLMC 9901	J.L.M. Charboneau 9901	ARIZ	USA, CA, Colusa Co.	23 Jul 2017
A. flexuosus (Hook.) Douglas ex G. Don var. *flexuosus*	*Scytocarpi*	JLMC 9674	J.L.M. Charboneau 9674	ARIZ	USA, NM, Otero Co.	31 Aug 2014
A. gypsodes Barneby	*Sarcocarpi*	MJM 3448	M.J. Moore 3448	OC	USA, NM, Eddy Co.	19 Aug 2016
A. lentiginosus Douglas var. *diphysus* (A. Gray) M.E. Jones	*Diphysi*	JLMC 9684	J.L.M. Charboneau 9684	ARIZ	USA, NM, San Juan Co.	25 May 2015
A. lentiginosus Douglas var. *mokiacensis* (A. Gray) M.E. Jones	*Diphysi*	JLMC 9866-1	J.L.M. Charboneau 9866	ARIZ	USA, UT, Washington Co.	18 May 2017
A. malacus A. Gray	*Malaci*	NO 511	N. Otting 511[a]	OSC	USA, OR, Malheur Co.	20 May 2003
A. mollissimus Torr. var. *mollissimus*	*Mollissimi*	MFW s.n.	M.F. Wojciechowski s.n.	ARIZ	USA, NM, Otero Co.	31 Aug 2014
A. neglectus (Torr. & A. Gray) E. Sheld.	*Neglecti*	DSA 2351	D.S. Anderson 2351[a]	MIN	USA, MN, Mahnomen Co.	28 Aug 2014
A. nuttallianus DC. var. *imperfectus* (Rydb.) Barneby	*Leptocarpi*	JLMC 9856	J.L.M. Charboneau 9856	ARIZ	USA, AZ, Pima Co.	4 Mar 2017
A. obscurus S. Watson	*Reventi-Arrecti*	HN 2002583	H. Nielsen 2002583[a]	OSC	USA, OR, Malheur Co.	20 Jun 2002

Supplementary Table S6 (continued)

Collections and Taxonomic Information for 25 Astragalus Taxa with Assembled Plastomes

Taxon	Section	Isolate	Voucher specimen	Herbarium	Locality	Date collected
A. pattersonii A. Gray	*Preussiani*	JLMC 9805-2	J.L.M. Charboneau 9805	ARIZ	USA, UT, Uintah Co.	27 May 2016
A. pectinatus (Hook.) Douglas ex G. Don	*Pectinati*	JLMC 9852-1	J.L.M. Charboneau 9852	ARIZ	USA, WY, Fremont Co.	24 Jun 2016
A. serenoi (Kuntze) E. Sheld. var. *serenoi*	*Nudi*	AT 16915	A. Tiehm 16915[a]	OSC	USA, NV, Churchill Co.	8 May 2015
A. tephrodes A. Gray var. *chloridae* (M.E. Jones) Barneby	*Argophylli*	JLMC 9741	J.L.M. Charboneau 9741	ARIZ	USA, AZ, Mohave Co.	29 Apr 2016
A. toanus M.E. Jones var. *toanus*	*Pectinati*	JLMC 9835-1	J.L.M. Charboneau 9835	ARIZ	USA, ID, Owyhee Co.	20 Jun 2016
A. wootonii E. Sheld. var. *wootonii*	*Inflati*	JLMC 9760	J.L.M. Charboneau 9760	ARIZ	USA, AZ, Yavapai Co.	7 May 2016

[a] DNA extracted from material on herbarium sheet; all others extracted from material dried in silica gel.

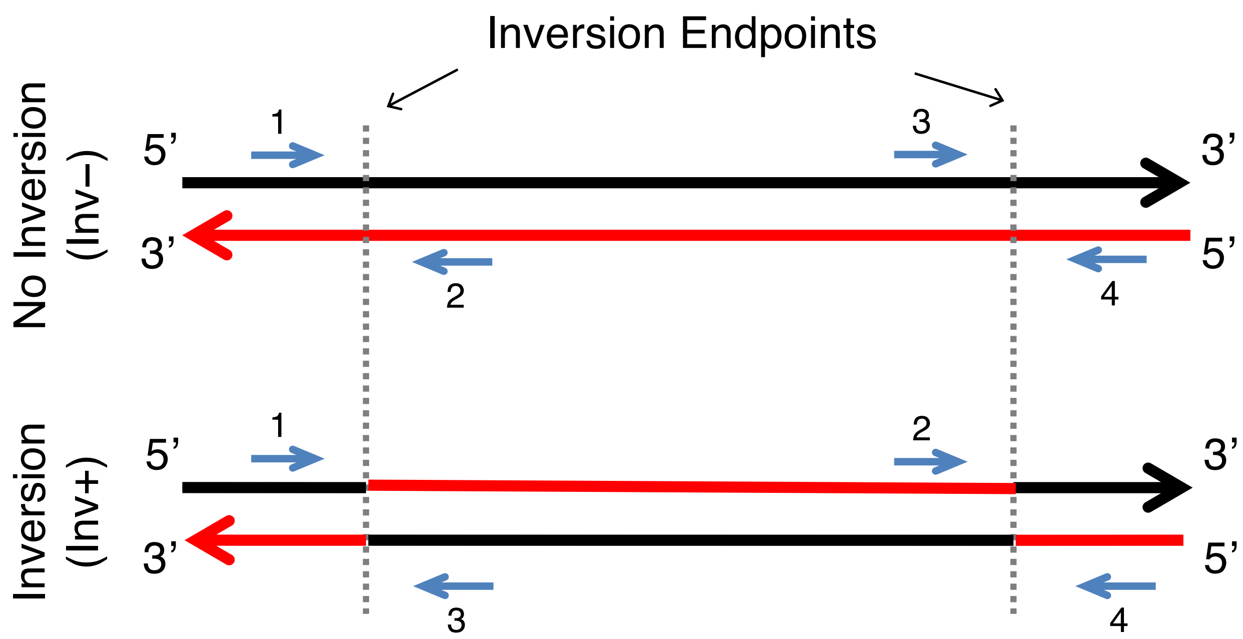

Supplementary Fig. S9.—Schematic of the positions of primers used to confirm the presence of plastome inversions. Inv– primer pairs (1 & 2, 3 & 4) should amplify when the inversion is not present, while Inv+ primer pairs (1 & 3, 2 & 4) should amplify when the inversion is present. Primers were designed within loci adjacent to inversion endpoints (see **supplementary table S7** for these loci for each inversion).

Supplementary Table S7

PCR Primers Used to Verify Presence of Plastome Inversions

Primer name	Inversion	Locus	Sequence
Acalyca-7kbInv1	*rbcL* ~ *trnH*-GUG	*ndhF*	5'-GGGAGAAGCTATCAAATATGTGGG-3'
Acalyca-7kbInv2	*rbcL* ~ *trnH*-GUG	*trnH*-GUG	5'-GGATCAAGGCAGTGGATTGTG-3'
Acalyca-7kbInv3	*rbcL* ~ *trnH*-GUG	*rbcL*	5'-GTTACTCGGAATGCTGCCAAG-3'
Acalyca-7kbInv4	*rbcL* ~ *trnH*-GUG	*atpB*	5'-CTAGACCTTTCACTACCAGAGCG-3'
Amoll-40kbInv1	*trnQ*-UUG ~ *trnT*-UGU	*trnL*-UAA	5'-ATTGCCCATTGTGAATTCCAGG-3'
Amoll-40kbInv2	*trnQ*-UUG ~ *trnT*-UGU	*trnT*-UGU	5'-GGAGTCGAACCGATGACCATC-3'
Amoll-40kbInv3	*trnQ*-UUG ~ *trnT*-UGU	*trnQ*-UUG	5'-GTAAGGCAACGGGTTTTGGTC-3'
Amoll-40kbInv4	*trnQ*-UUG ~ *trnT*-UGU	*accD*	5'-CCATAGGATTCCACGTACCTGG-3'
Aflex-7kbInv1	*trnL*-CAA ~ *trnI*-CAU	*rpl23*	5'-CTGTTCTAGTTGATCCCGATTCG-3'
Aflex-7kbInv2	*trnL*-CAA ~ *trnI*-CAU	*trnM*-CAU	5'-GCCCAACTCATAATTGGCGAAG-3'
Aflex-7kbInv3	*trnL*-CAA ~ *trnI*-CAU	*trnL*-CAA	5'-GCGTGTCTACCATTTCACCAC-3'
Aflex-7kbInv4	*trnL*-CAA ~ *trnI*-CAU	*ndhB*	5'-CCCCATTATTGAAATTGCTCAGG-3'
trnL-UAA-intronF[a]	(positive control)	*trnL*-UAA exon 1	5'-TGGCGAAATAGGTAGACGCTAC-3'
trnL-UAA-intronR[a]	(positive control)	*trnL*-UAA exon 2	5'-GGACTTGAACCCTCACGATTTC-3'

[a]Based on sequences of universal primers in Taberlet et al. (2007).

Supplementary Table S8

Annealing Temperatures and Extension Times Used in PCR to Verify the Presence of Plastome Inversions

Inversion	Annealing temp. (°C)	Extension time (s)
rbcL ~ *trnH*-GUG	53	70
trnQ-UUG ~ *trnT*-UGU	53	150
trnL-CAA ~ *trnI*-CAU	52	60
(positive control)	53	45

Supplementary Material Literature Cited

Altschul SF, Gish W, Miller W, Myers EW, Lipman DJ. 1990. Basic local alignment search tool. J Mol Biol. 215(3):403–410.

Benson G. 1999. Tandem repeats finder: a program to analyze DNA sequences. Nucleic Acids Res. 27(2):573–580.

Boisvert S, Laviolette F, Corbeil J. 2010. Ray: simultaneous assembly of reads from a mix of high-throughput sequencing technologies. J Comp Biol. 17(11):1519–1533.

Bolger AM, Lohse M, Usadel B. 2014. Trimmomatic: a flexible trimmer for Illumina sequence data. Bioinformatics. 30(15):2114–2120.

Bushnell B, Rood J, Singer E. 2017. BBMerge – accurate paired shotgun read merging via overlap. PLoS One. 12(10):e0185056.

Hoang DT, Chernomor O, von Haeseler A, Minh BQ, Vinh LS. 2017. UFboot2: improving the ultrafast bootstrap approximation. Mol Biol Evol. 35(2):518–522.

Kalyaanamoorthy S, Minh BQ, Wong TKF, von Haeseler A, Jermiin LS. 2017. ModelFinder: fast model selection for accurate phylogenetic estimates. Nat Methods. 14(6):587–589.

Kim K-J, Choi K-S, Jansen RK. 2005. Two chloroplast DNA inversions originated simultaneously during the early evolution of the sunflower family (Asteraceae). Mol Biol Evol. 22(9):1783–1792.

Laslett D, Canback B. 2004. ARAGORN, a program to detect tRNA genes and tmRNA genes in nucleotide sequences. Nucleic Acids Res. 32(1):11–16.

Lee HL, Jansen RK, Chumley TW, Kim KJ. 2007. Gene relocations within chloroplast genomes of *Jasminum* and *Menodora* (Oleaceae) are due to multiple, overlapping inversions. Mol Biol Evol. 24(5):1161–1180.

Li H, Durbin R. 2010. Fast and accurate long-read alignment with Burrows-Wheeler transform. Bioinformatics. 26(5):589–595.

Lowe TM, Eddy SR. 1997. tRNAscan-SE: a program for improved detection of transfer RNA genes in genomic sequence. Nucleic Acids Res. 25(5):955–964.

Minh BQ, et al. 2020. IQ-TREE 2: new models and efficient methods for phylogenetic inference in the genomic era. Mol Biol Evol. 37(5):1530–1534.

Nikolenko SI, Korobeynikov AI, Alekseyev MA. 2013. BayesHammer: Bayesian clustering for error correction in single-cell sequencing. BMC Genom. 14(Suppl 1):57.

Pagel M. 1999. Inferring the historical patterns of biological evolution. Nature. 401:877–884.

Paradis E, Schliep K. 2019. ape 5.0: an environment for modern phylogenetics and evolutionary analyses in R. Bioinformatics. 35(3):526–528.

Pinheiro J, Bates D, Sarkar D, R Core Team. 2020. nlme: linear and nonlinear mixed effects models. Vienna (Austria): Comprehensive R Archive Network (CRAN).

Qu X-J, Moore MJ, Li D-Z, Yi T-S. 2019. PGA: a software package for rapid, accurate, and flexible batch annotation of plastomes. Plant Methods. 15:50.

Quinlan AR, Hall IM. 2010. BEDTools: a flexible suite of utilities for comparing genomic features. Bioinformatics. 26(6):841–842.

R Core Team. 2020. R: a language and environment for statistical computing. Vienna (Austria): R Foundation for Statistical Computing.

Revell LJ. 2012. phytools: an R package for phylogenetic comparative biology (and other things). Methods Ecol Evol. 3(2):217–223.

Taberlet P, et al. 2007. Power and limitations of the chloroplast *trnL* (UAA) intron for plant DNA barcoding. Nucleic Acids Res. 35(3):e14.

Tillich M, et al. 2017. GeSeq – versatile and accurate annotation of organelle genomes. Nucleic Acids Res. 45:W6–W11.

Van Dongen S. 2002. Graph clustering by flow simulation [PhD Thesis]. Utrecht (Netherlands): University of Utrecht.

Wheeler TJ, Eddy SR. 2013. nhmmer: DNA homology search with profile HMMs. Bioinformatics. 29(19):2487–2489.
